# *Stenotrophomonas rhizophila* Ep2.2 inhibits growth of *Botrytis cinerea* through the emission of volatile organic compounds, restricts leaf infection and primes defense genes

**DOI:** 10.3389/fpls.2023.1235669

**Published:** 2023-10-02

**Authors:** Aida Raio, Federico Brilli, Luisa Neri, Rita Baraldi, Francesca Orlando, Claudio Pugliesi, Xiaoyulong Chen, Ivan Baccelli

**Affiliations:** ^1^Institute for Sustainable Plant Protection (IPSP), National Research Council of Italy (CNR), Florence, Italy; ^2^Institute for BioEconomy (IBE), National Research Council of Italy (CNR), Bologna, Italy; ^3^Department of Agriculture, Food and Environment, University of Pisa, Pisa, Italy; ^4^College of Agriculture, College of Tobacco Science, Guizhou University, Guiyang, China

**Keywords:** beneficial microbes, biological control agents (BCAs), *Solanum lycopersicum*, plant pathogens, plant microbiome, induced resistance, defense priming, antimicrobial VOCs

## Abstract

The bacterium *Stenotrophomonas rhizophila* is known to be beneficial for plants and has been frequently isolated from the rhizosphere of crops. In the present work, we isolated from the phyllosphere of an ornamental plant an epiphytic strain of *S. rhizophila* that we named Ep2.2 and investigated its possible application in crop protection. Compared to *S. maltophilia* LMG 958, a well-known plant beneficial species which behaves as opportunistic human pathogen, *S. rhizophila* Ep2.2 showed distinctive features, such as different motility, a generally reduced capacity to use carbon sources, a greater sensitivity to fusidic acid and potassium tellurite, and the inability to grow at the human body temperature. *S. rhizophila* Ep2.2 was able to inhibit *in vitro* growth of the plant pathogenic fungi *Alternaria alternata* and *Botrytis cinerea* through the emission of volatile compounds. Simultaneous PTR-MS and GC-MS analyses revealed the emission, by *S. rhizophila* Ep2.2, of volatile organic compounds (VOCs) with well-documented antifungal activity, such as furans, sulphur-containing compounds and terpenes. When sprayed on tomato leaves and plants, *S. rhizophila* Ep2.2 was able to restrict *B. cinerea* infection and to prime the expression of *Pti5*, *GluA* and *PR1* plant defense genes.

## 1 Introduction

Plant-associated microbes that collectively constitute the plant microbiota can live inside plants as endophytes, or populate as epiphytes on the surface of roots, leaves and other organs (Bulgarelli et al., 2013; Berg et al., 2014). The plant microbiota affects deeply plant growth, productivity and resistance to stresses (Berg et al., 2017). However, it has been demonstrated that crops may display reduced microbial diversity as compared to their wild relatives, probably because this trait was impoverished by the domestication process to which they were subjected (Martínez-Romero et al., 2020). For these reasons, the plant microbiota has become the central target of emerging biotechnological strategies aimed at improving yields and resilience of crops: microbiome engineering, microbiome management and microbiome-based products are believed to represent promising alternatives to reduce chemical inputs in agriculture (Berg et al., 2017; Arif et al., 2020; Ke et al., 2021; Malacrinò et al., 2022). The plant microbiota is a precious source of novel beneficial microbes that may be used in agriculture as biofertilizers, biostimulants, biological control agents (BCAs) (El-Saadony et al., 2022), or as source of microbes that may be assembled in artificial (synthetic) consortia to reconstruct the structure and function of impaired plant microbiomes (Arif et al., 2020).

Among the microbes possessing the ability to improve plant performance and health, plant growth-promoting rhizobacteria (PGPR) are probably the most known category. PGPR can increase the availability of nutrients and synthesize phytohormones thereby promoting plant growth, or they may produce antimicrobial compounds and prime immune responses thereby improving resistance to pathogens (Mauch-Mani et al., 2017; Tabassum et al., 2017; El-Saadony et al., 2022). Volatile organic compounds (VOCs) emitted by beneficial microbes can affect the mechanisms of plant tolerance to abiotic (Liu and Zhang, 2015; Brilli et al., 2019) and biotic stresses (Enebe and Babalola, 2019; Thankappan et al., 2022). For instance, VOCs emitted by PGPR can inhibit the growth of plant pathogenic fungi and bacteria either directly (Raio et al., 2020) or indirectly by activating plant defenses (Brilli et al., 2019; Liu and Brettell, 2019). Studies have also shown how VOCs emitted by a single bacterial strain can simultaneously inhibit pathogen growth and induce plant defense (Sharifi and Ryu, 2016).

Therefore, to reduce our dependence on agrochemicals, the identification, isolation and characterization of novel plant-associated beneficial microbes is strongly demanded. In the present work, we have investigated a novel strain of the Gram-negative bacterium *Stenotrophomonas rhizophila* that we isolated from the phyllosphere of *Hibiscus syriacus* plants and named Ep2.2.

*S. rhizophila* belongs to the class of *Gammaproteobacteria*, order *Xanthomonadales*, family *Xanthomonadaceae*. The genus *Stenotrophomonas* was first described for the species *S. maltophilia*, formerly known as *Pseudomonas maltophilia* and subsequently *Xanthomonas maltophilia* (Palleroni and Bradbury, 1993). *S. maltophilia* was used as an efficient biocontrol agent for a long time, until it was found to behave as an opportunistic human pathogen in immunocompromised patients (Ryan et al., 2009; Berg and Martinez, 2015). For this reason, *S. rhizophila* has attracted increasing attention in recent years as a harmless alternative for biotechnological applications (Berg and Martinez, 2015). *S. rhizophila* was identified for the first time in the rhizosphere of rape and potato plants (Wolf et al., 2002) and reported to colonize roots behaving as endophyte in plants (Berg and Martinez, 2015). However, *S. rhizophila* appears to be a ubiquitous bacterium, since isolates have been collected not only from plants but also from very different environments, such as marine environments and underground archeological sites (Rivas-Garcia et al., 2019; Cuzman et al., 2023). *S. rhizophila* possesses plant growth-promoting ability and biocontrol properties against phytopathogens, but its mode of action has often remained elusive (Kai et al., 2007; Ryan et al., 2009; Schmidt et al., 2012; Maurer et al., 2013; Reyes-Perez et al., 2019; Rivas-Garcia et al., 2019).

Here, the phyllosphere epiphytic strain Ep2.2 of *S. rhizophila* was characterized biochemically and metabolically in comparison to *S. maltophilia*, and its biocontrol activity against different fungal plant pathogens was assessed. By coupling Proton Transfer Reaction – Quadrupole Mass Spectrometer (PTR-MS) and Gas Chromatography–Mass Spectrometry (GC-MS) analyses, we thoroughly analyzed *in vitro* the complex blend of VOCs produced by *S. rhizophila* Ep2.2, in order to identify those having antifungal activity. Then, we analyzed the ability of *S. rhizophila* Ep2.2 to restrict *B. cinerea* infection on tomato leaves and to prime plant defense genes.

## 2 Materials and methods

### 2.1 Isolation and identification of the epiphytic strain Ep2.2 of *Stenotrophomonas rhizophila*

*Hibiscus syriacus* plant samples were collected during a survey on the cultivable bacterial population inhabiting ornamental plants. The survey was carried out in 2012 in a commercial nursery located in Pistoia, Italy, by sampling one plant per species. Ten grams of leaves, stems and buds were suspended in 90 mL of a solution containing 1% peptone and 1% Tween 90, and shaken at 200 rpm for one hour at room temperature. Three suspensions were prepared, bulked and then spread as 100 µL aliquots onto the surface of nutrient glucose agar (NGA) medium amended with cycloheximide (200 ppm). Plates were incubated at 25 ± 2°C for one week, after that bacterial colonies of different morphology were picked up and streaked (at least twice) on NGA medium for purification. Pure cultures were suspended in 30% glycerol solution and maintained at -80°C until this work was carried out.

The *S. rhizophila* strain that we named Ep2.2 was identified by 16S rDNA amplification with the primers fD1 (5’–GAGTTTGATCCTGGCTCAG–3’) and rP1/rP2 (5’– GGYTACCTTGTTACGACTT–3’; Y=C/T) (Pious and Thyvalappil, 2009) according to the protocol described by Krimi et al. (2016). The amplified 16S rDNA fragment was analyzed for similarity by comparing to known nucleotide sequences present in the NCBI GenBank database by BLASTn search (http://blast.ncbi.nlm.nih.gov/). The taxonomical affiliation was also determined by aligning the *S. rhizophila* Ep2.2 16S rDNA sequence to 16S rDNA sequences available in the NCBI database belonging to six *Stenotrophomonas* species, three *Xanthomonas* species, and *Xylella fastidiosa*. The evolutionary history was inferred using the Neighbor-Joining method (Saitou and Nei, 1987). The evolutionary distances were computed using the Maximum Composite Likelihood method (Tamura et al., 2004) and expressed in the units of the number of base substitutions per site. Evolutionary analyses were conducted in MEGA 6 (Tamura et al., 2013). The *S. rhizophila* Ep2.2 16S rDNA sequence was deposited in the EMBL/GenBank/DDBJ nucleotide databases under the accession number MZ841807. *S. rhizophila* Ep2.2 is included in the microbial collection of the IPSP-CNR (Sesto Fiorentino, Italy).

### 2.2 Biochemical and metabolic characterization of *Stenotrophomonas rhizophila* Ep2.2

*S. rhizophila* Ep2.2 was assessed for Gram reaction by the KOH test (Buck, 1982), for catalase and oxidase activities (Schaad et al., 2001), for siderophore production on King agar B medium (Sigma-Aldrich, MO, USA) following the procedure of Mikiciński et al. (2016), and for glucanase activity on tryptic soy agar medium (Sigma-Aldrich, MO, USA) amended with glucane 0.1%. Proteolytic, lipolytic and chitinolytic activity tests were performed on skim milk agar, LB agar (Sigma-Aldrich, MO, USA) amended with 1% Tween 40, and Chitin azure medium (Sigma-Aldrich, MO, USA), respectively. Exopolysaccharide (EPS) production was assayed by using the protocol described by Tallgren et al. (1999). The ability to grow at 4, 30, 37 and 40°C was assessed in LB medium after one week of incubation. 1-aminocyclopropane-1-carboxylic acid (ACC) deaminase activity was detected by means of M9 minimal medium with ACC as unique N source (Penrose and Glick, 2003). The ability to solubilize P was verified by using the NPRBB growth medium (Nautiyal, 1999). Indole-3-acetic acid (IAA) production was tested on LB medium amended with L-triptophan according to Bric et al. (1991). The bacterial strains used as reference for the different biochemical tests are reported in **Supplementary Table S1**.

Swimming, swarming and twitching motility, and ability to form biofilm, were determined according to Déziel et al. (2001). Motility was assessed in comparison to *S. maltophilia* LMG 958, *Xanthomonas campestris* pv. *campestris* Xcc1*, Erwinia amylovora* E1 and *Agrobacterium tumefaciens* C58. The biofilm production assay was modified to be performed in microtiter plates (Costar assay plate, Corning, NY, USA).

The metabolic profile of *S. rhizophila* Ep2.2 was analyzed with the BIOLOG system by using GEN III MicroPlate (Catalog No. 1030, Rigel, Italy), according to the protocol provided by the manufacturer (BIOLOG, USA). The GEN III MicroPlate includes 94 phenotypic tests: 71 carbon source utilization assays and 23 chemical sensitivity assays, and allows identifying bacteria at the species level. The analysis of the metabolic fingerprint was performed by incubating at 30°C for 75 h in the OmniLog device (BIOLOG, USA), which yields colorimetric curves indicating utilization of the carbon sources or resistance to inhibitory chemicals, as a result of cell respiration. Respiration causes reduction of a tetrazolium redox dye and formation of purple color. The test was carried out in comparison with *S. maltophilia* type strain LMG 958 in order to highlight metabolic differences with *S. rhizophila* Ep2.2.

### 2.3 *In vitro* estimation of the antagonistic activity of *Stenotrophomonas rhizophila* Ep2.2 against fungal plant pathogens

The antagonistic activity of *S. rhizophila* Ep2.2 was evaluated by dual culture assay against four fungal plant pathogens: *Alternaria alternata*, *Botrytis cinerea*, *Fusarium oxysporum* f. sp. *lycopersici* and *Rhizoctonia solani*. Briefly, 5 mm plugs were cut from the margin of fresh fungal colonies grown on potato dextrose agar (PDA; VWR Chemicals, Belgium) medium and placed in the middle of Petri dishes containing the same medium. Two 50 µL aliquots of *S. rhizophila* Ep2.2 suspension (OD_600 nm_ = 0.1, corresponding to 1x10^8^ cells/mL) were streaked at the two opposite sides of the fungal plug. The plates were incubated at 26°C for six days before measuring fungal colony diameters and comparing to control cultures grown in the absence of *S. rhizophila* Ep2.2.

Subsequently, to demonstrate the inhibitory role of volatile compounds produced by *S. rhizophila* Ep2.2 on *B. cinerea* growth, Petri dishes, prepared as described above, were either sealed with three layers of parafilm or kept unsealed, and incubated at 26°C for 2, 3 or 7 days. Diameters of fungal colonies grown both on sealed and unsealed plates were then measured and compared.

In order to exclude inhibition by diffusible molecules, a culture filtrate of *S. rhizophila* Ep2.2 was prepared and tested against *B. cinerea* as described below. *S. rhizophila* Ep2.2 was grown on Nutrient Broth (Scharlab S.L., Spain) amended with 2.5 g/L glucose (NGB) on an orbital shaker at 25 ± 2°C, 120 rpm, for 48 h. The bacterial suspension was then centrifuged at 10,000 rpm for 10 min. and the supernatant was then collected and sterilized through a Millipore 0.2 µm filter. The sterile filtrate (1.5 mL) was mixed with 15 mL of PDA cooled at 55°C. After medium solidification, a 0.5 mm agar plug was cut from the margin of a colony of *B. cinerea* grown on PDA and placed in the middle of the Petri dish. The test was conducted in triplicate. *B. cinerea* was grown on unamended PDA as a control. Plates were incubated at 26°C for 4 days after that the inhibitory activity was evaluated by measuring the diameter of *B. cinerea* colonies.

### 2.4 *In vitro* assessment of the inhibitory activity of volatile compounds from *Stenotrophomonas rhizophila* Ep2.2 on the growth of phytopathogenic fungi

Two-compartment 92-mm-diameter Petri dishes with ventilation cams and common headspace (Sarstedt, Nümbrecht, Germany) were used to determine the inhibiting activity of volatile compounds produced by *S. rhizophila* Ep2.2 against the four different phytopathogenic fungi listed in the previous paragraph. An aliquot (7 mL) of agarized NGB medium (NGA) was poured into one of the two compartments of the plate, while 7 mL of PDA medium were poured into the other compartment. Once dried, 50 μL of *S. rhizophila* Ep2.2 bacterial suspension (OD_600 nm_ = 0.1) were spread on NGA medium, whereas a 5-mm diameter plug was cut from the edge of the fungal colony grown on PDA and placed at the center of the other compartment containing PDA. As a control, two-compartment plates containing non-inoculated NGA and fungus-inoculated PDA were used. Inoculated plates were sealed with 3 layers of parafilm to prevent dispersion of volatile compounds, and the inhibitory activity on phytopathogenic fungi was evaluated by measuring the colony diameter after 3 days of incubation at 26°C. Four replicates per each *S. rhizophila* Ep2.2-fungus combination were analyzed.

### 2.5 *In vitro* analysis of volatile organic compounds (VOCs) emitted by *Stenotrophomonas rhizophila* Ep2.2

#### 2.5.1 Bacterial culture preparation

*S. rhizophila* Ep2.2 was grown on NGA plates at 26°C for 48 h. Bacterial cells were scraped from the agar surface and suspended in 0.8% NaCl to obtain 1x10^8^ CFU mL^−1^ suspensions (OD_600 nm_ = 0.1). An aliquot of 500 μL of each suspension was added to 500 mL airtight flasks containing 50 mL of NGA medium. All the flasks were then incubated at 26°C for 48 h before PTR-MS and GC-MS analyses.

#### 2.5.2 PTR-MS analysis

Emission of VOCs from *S. rhizophila* Ep2.2 was screened in real-time by PTR-MS through direct air sampling of the flask headspace above the bacterial culture, with a PTR-MS instrument (Ionicon Analytic GmbH, Innsbruck, Austria). In particular, VOCs were detected following chemical ionization between molecules of H_3_O^+^ (produced at high density in an ion source) and those of VOCs present into the headspace air and having a proton affinity higher than that of H_2_O (= 166 kcal mol^-1^). Proton transfer reaction occurred in a drift tube under constant conditions of pressure (= 2.2 mbar), temperature (= 50°C) and electrical field (600 v cm^-2^), thus resulting in an ionization energy E/N = 130 Td (Lindinger et al., 1998). All the protonated ions related to VOCs and/or fragment of VOCs were analyzed with a duty cycle of 200 s spanning from *m/z* 20 to 220 *m/z* 20 with a dwell time = 1 s for each single *m/z*. An amount of 6 full cycles were completed for each analyzed flask, during which the 100 mL min^-1^ of the headspace air was sampled by PTR-MS and simultaneously replaced within the flasks with the same amount of VOC-free air produced by a customized zero air generator. All the 6 cycles recorded from one flask headspace were averaged within one single measurement, and the different measurements were replicated nine times in two independent experiments. In addition, the headspace of four flasks containing only NGA medium were measured by PTR-MS and the resulting averaged value was subtracted to those of the flasks containing *S. rhizophila* Ep2.2 (Brilli et al., 2019; Raio et al., 2020).

#### 2.5.3 GC-MS analysis

An external pump (Pocket Pump SKC Inc., PA, USA) was used to sample 100 mL of the flask headspace air above the *S. rhizophila* Ep2.2 culture at a flow rate of 50 mL min^−1^ in a cartridge filled with 200 mg of Tenax GC^®^ (Markes International, Ltd, Llantrisant, UK). After sampling, all the cartridges were thermally-desorbed for 15 min at 280°C with a helium flow rate of 50 mL min^−1^ (Markes International, Series 2 Unity) and VOCs were transferred into a cold trap rapidly heated from 10°C to 280°C. Subsequently, VOCs were separated and further identified with a 7890A gas chromatograph coupled with a 5975C mass detector (GC–MS, Agilent Technologies, Wilmington, USA) through fast injection onto a capillary column (ZB-1, 60 m × 0.25 mm I.D. × 0.25 μm film of polymethylsiloxane; Phenomenex, Inc. Torrance, CA, USA) via a transfer line heated at 200°C. In particular, peak integration and identification of VOCs were performed through ChemStation software (Agilent Technologies, Wilmington, USA) through comparison of the retention times and the fragmentation patterns listed in the NIST11 database of mass spectra. Furthermore, the identified VOCs were quantified by using an external standard calibration procedure obtained by means of calibrated gas cylinders of different VOCs (Rapparini et al., 2004; Baraldi et al., 2019).

### 2.6 Ability of *Stenotrophomonas rhizophila* Ep2.2 to protect tomato leaves against *Botrytis cinerea* infection

*Solanum lycopersicum* cv. Micro-Tom and cv. Marmande plants were grown in a growth room under LED lights (photoperiod 12/12 h) as previously described (Baccelli et al., 2022). The ability of *S. rhizophila* Ep2.2 to protect from *B. cinerea* infection was first tested on Micro-Tom leaves detached from plants during their second month of growth. A number of 10-13 mature and healthy leaves (selected among the 3^rd^ to the 5^th^ leaf) were cut from different undamaged plants and placed into 90 mm-Petri dishes containing a filter paper disc soaked with 1 mL sterile water to ensure high internal relative humidity (RH) conditions during incubations. *S. rhizophila* Ep2.2 was grown overnight in NGB medium at 28°C, centrifuged at low speed, washed twice in 10 mM MgCl_2_, and finally suspended in 10 mM MgCl_2_ (OD_600 nm_ = 0.1) for leaf treatment. Treatments were performed by spraying the lower (abaxial) surface of a leaf with approximately 300 μL of bacterial suspension with a 10-mL pump atomizer. Control leaves were sprayed with 10 mM MgCl_2_. All the plates were sealed with parafilm and incubated at 21°C (day)/18°C (night), photoperiod 12/12 h (100 μmol m^2^ s^−1^) for 48 h, after that *B. cinerea* strain B05.10 was inoculated. For pathogen inoculation, conidia were collected from 15-20 day-old *B. cinerea* cultures grown in PDA under a light/dark regime (Schumacher, 2017; Baccelli et al., 2022). Conidia were suspended in potato dextrose broth (PDB, Laboratorios Conda S.A., Spain) at the concentration of 1x10^6^ conidia/mL and inoculated on leaves by applying one, or two, 10-µL droplets on each side of the midrib on the abaxial leaf surface. All the plates were sealed once again with parafilm and incubated as described above. Necrotic lesions caused by *B. cinerea* infection were measured after 3 days of incubation. The protective effect shown by *S. rhizophila* Ep2.2 against *B. cinerea* was validated on whole plants by using the tomato cv. Marmande. Plants were sprayed 2 weeks after germination on the adaxial leaf surfaces with *S. rhizophila* Ep2.2 as described above, incubated 48 h under high RH conditions, and subsequently inoculated on the adaxial surface of two opposite leaves (2^nd^ and 3^rd^) with a drop of a conidial suspension prepared as already described. Lesions were measured after 3 days of incubation.

### 2.7 Gene expression analyses in tomato leaves treated with *Stenotrophomonas rhizophila* Ep2.2

The expression of plant defense genes was analyzed by RT-qPCR to investigate the ability of *S. rhizophila* Ep2.2 to induce localized resistance in leaves. The analyses were designed to reveal either the ability of *S. rhizophila* Ep2.2 to induce directly defense genes before infection, or prime them for a quicker/boosted induction during *B. cinerea* B05.10 infection. Leaves from different 5-week-old *S. lycopersicum* cv. Micro-Tom plants were detached and treated as follows: a) leaves were spray-treated on their abaxial surface either with *S. rhizophila* Ep2.2 (OD_600 nm_ = 0.1 in 10 mM MgCl_2_), or with 10 mM MgCl_2_ as control, and then incubated for 48 h (samples named as “*S. rhizophila*” and “control”, respectively, at 48 hours post treatment, hpt); b) leaves were spray-treated on their abaxial surface with either *S. rhizophila* Ep2.2, or 10mM MgCl_2_ as control, incubated for 48 h, and subsequently inoculated with 10-µL droplets (3-6 per leaf) of 1x10^6^ *B. cinerea* conidia/mL in PDB (samples named as “*S. rhizophila* + *B.cinerea*” and “control + *B. cinerea*”, respectively, at 6 or 24 h post infection, hpi); c) leaves were spray-treated on their abaxial surface with either *S. rhizophila* Ep2.2, or 10 mM MgCl_2_ as control, incubated for 48 h, and subsequently mock inoculated with 10-µL droplets (3-6 per leaf) of PDB (samples named as “*S. rhizophila* + mock” and “control + mock”, respectively, at 6 and 24 hpi). Each biological replicate consisted of two leaves belonging to different plants, and three biological replicate per condition were prepared. Leaves were incubated into 90 mm-Petri dishes as described in the previous paragraph and frozen in liquid nitrogen upon sampling. To check that the treatments were leading to the expected reduction in disease symptoms, some “control + *B. cinerea*” and “*S. rhizophila* + *B. cinerea*” leaves were inoculated with a single 10-µL drop of conidial suspension prepared as described above and kept incubating for a longer period (96 hpi). Lesion diameters were then measured.

For RNA extraction, leaves were ground in liquid nitrogen and total RNA was extracted by using RNeasy Plant Mini Kit with buffer RLT (Qiagen, Italy) (Baccelli et al., 2022). The extracted RNA was quantified in a Qubit fluorometer (Thermo Fisher Scientific, MA, USA) and its integrity verified by agarose gel electrophoresis. Amplification Grade DNase I (Sigma-Aldrich) was used to degrade traces of contaminating DNA before reverse-transcription, which was performed with Maxima First Strand cDNA Synthesis Kit (Thermo Fisher Scientific, MA, USA). qRT-PCRs were performed in a StepOne Real-Time PCR System (Applied Biosystems, Thermo Fisher Scientific Inc. Waltham, MA, USA) by using Fast SYBR™ Green Master Mix (Applied Biosystems, Vilnius, Lithuania) as described in Baccelli et al. (2022).

The following genes were analyzed: *Proteinase inhibitor II* (*PIN2*), *β-1,3-glucanase A* (*GluA*), *Pathogenesis-related protein 1* (*PR1*), *1-aminocyclopropane- 1-carboxylate oxidase 1* (*ACO1*), *Pathogenesis-related genes transcriptional activator* (*PTI5*), *Lipoxygenase A* (*Lox1.1*), and *Polygalacturonase inhibitor protein* (*PGIP*). The genes were selected based on their known modulation occurring during *B. cinerea* infection in tomato cv. Micro-Tom (Baccelli et al., 2022). Gene locus IDs and primer sequences are reported in Baccelli et al. (2022). Relative gene expression values were calculated by using the 2^-ΔΔ^*^C_T_^* method as described in Livak and Schmittgen (2001) after melting curve analysis and amplification plot comparisons. *Actin-7* was used as the endogenous reference gene for transcript normalization (Baccelli et al., 2022). Three biological replicates and two technical replicates were analyzed per each condition.

### 2.8 Statistical analyses

Motility of bacterial strains was analyzed by one-way ANOVA with Tukey–Kramer multiple comparison post-test (*p* ≤ 0.05) by performing the analysis separately per each time point. Data concerning colony and lesion diameters were analyzed by unpaired *t*-test (*S. rhizophila*-treated vs. control) and considered significant at *p* ≤ 0.05. Relative gene expression values were analyzed by unpaired *t*-test (48 hpt) or one-way ANOVA with Tukey–Kramer multiple comparison post-test (6 and 24 hpi) after normality check, and considered significantly different at *p* ≤ 0.05. Analyses were performed in GraphPad Prism 9 (GraphPad Software Inc., CA, USA).

## 3 Results

### 3.1 Identification and characterization of the epiphytic strain Ep2.2 of *Stenotrophomonas rhizophila*

**Figure 1:**
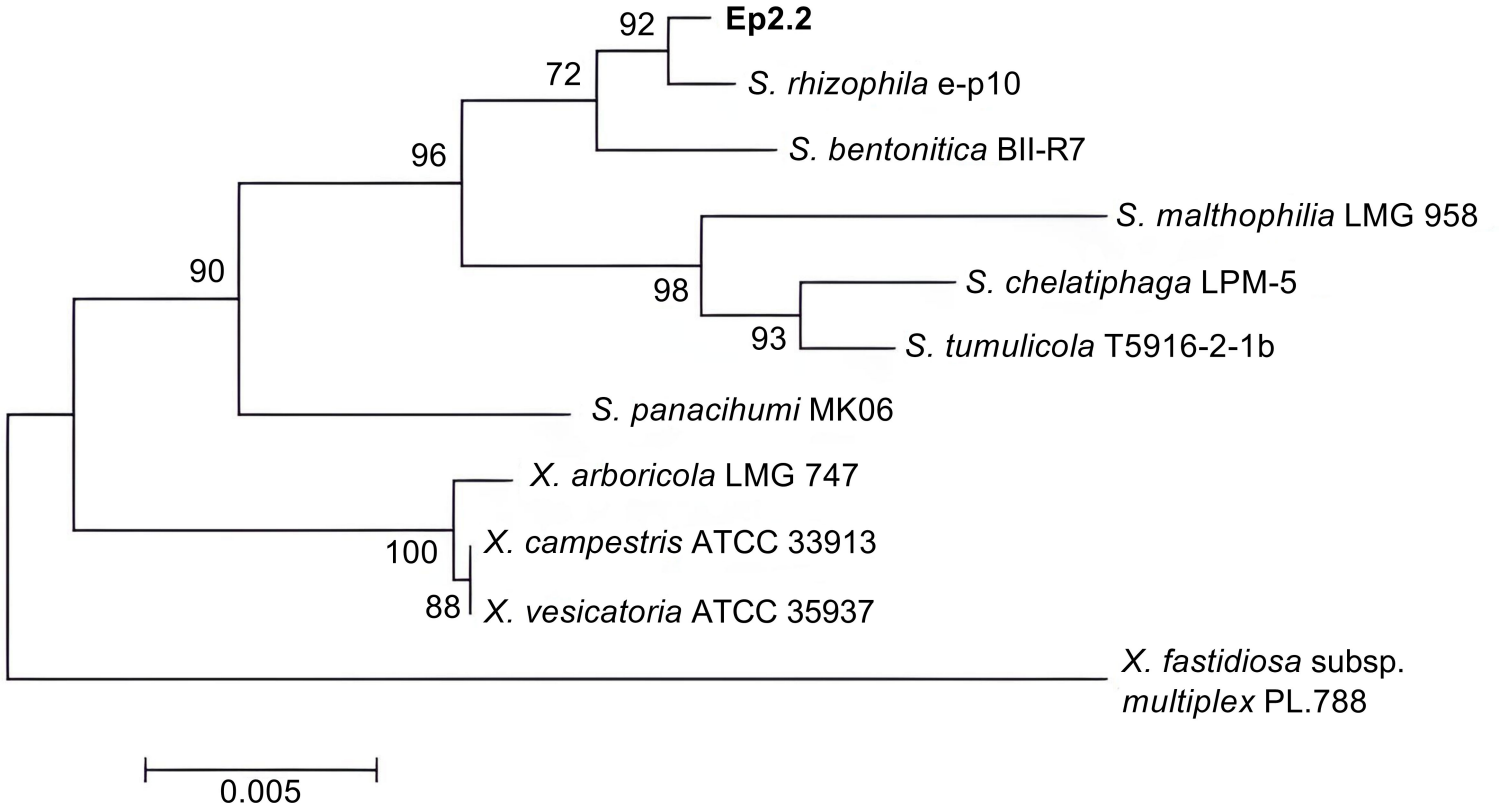
Phylogenetic tree showing the evolutionary relationships of the strain Ep2.2 identified by 16S rDNA sequencing as *Stenotrophomonas rhizophila*. The neighbor-joining analysis was performed by using 16S rDNA sequences available in the NCBI database: *Stenotrophomonas rhizophila* e-p10 (acc. no. NR_121739.1), *Stenotrophomonas bentonitica* BII-R7 (acc. no. NR_157765.1), *Stenotrophomonas malthophilia* LMG 958 (acc. no. NR_119220.1), *Stenotrophomonas chelatiphaga* LPM-5 (acc. no. NR_116366.1), *Stenotrophomonas tumulicola* T5916-2-1b (acc. no. NR_148818.1), *Stenotrophomonas panacihumi* MK06 (acc. no. NR_117406.1), *Xanthomonas arboricola* LMG 747 (acc. no. NR_125714.1), *Xanthomonas campestris* ATCC 33913 (acc. no. NR_074936.1), *Xanthomonas vesicatoria* ATCC 35937 (acc. no. NR_026388.1), and *Xylella fastidiosa* subsp. *multiplex* PL.788 (acc. no. NR_041783.1) (outgroup). The optimal tree with the sum of branch length = 0.08281655 is shown. The percentage of replicate trees in which the associated taxa clustered together in the bootstrap test (1000 replicates) are shown next to the branches (Felsenstein, 1985). The tree is drawn to scale, with branch lengths in the same units as those of the evolutionary distances used to infer the phylogenetic tree.

The bacterial strain that we named Ep2.2 was isolated from aboveground organs of a *H. syriacus* plant, as described in materials and methods. The analysis of 16S rDNA sequence (GenBank acc. no. MZ841807) allowed identifying the strain as *S. rhizophila* (99.78% nucleotide identity). The phylogenetic analysis clustered Ep2.2 with the reference *S. rhizophila* strain e-p10, clearly separating them from other *Stenotrophomonas* and *Xanthomonas* species (**Figure 1**).

**Figure 2:**
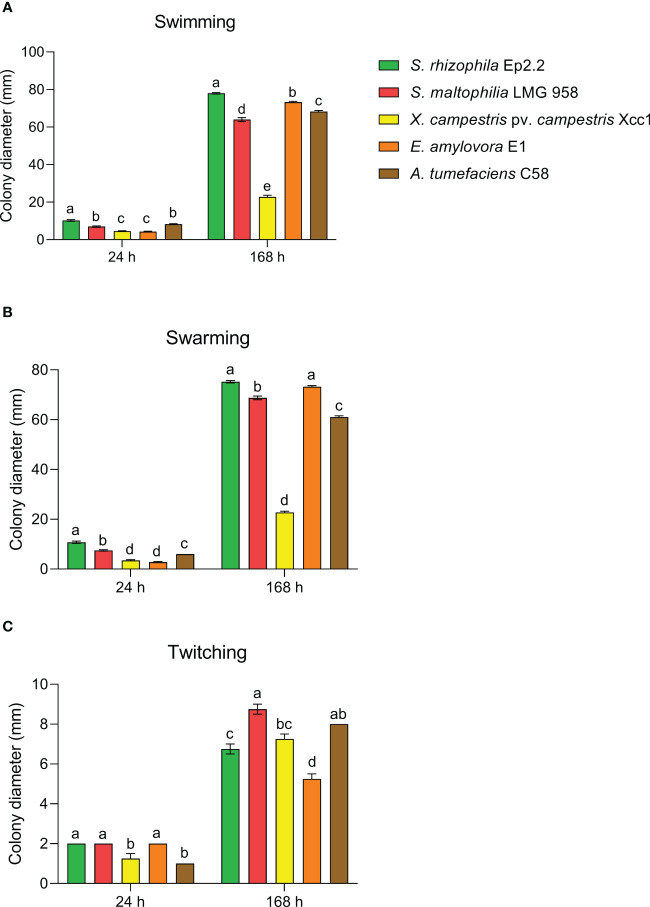
Motility of *Stenotrophomonas rhizophila* strain Ep2.2. Swimming **(A)**, swarming **(B)**, and twitching **(C)** abilities were compared to *S. maltophilia*, *Xanthomonas campestris* pv. *campestris, Erwinia amylovora* and *Agrobacterium tumefaciens*. Colony diameters (mean ± SEM, *n* = 4) were measured after 24 and 168 hours (i.e. 7 days) of incubation at 26°C. Different letters indicate statistically significant differences within each time point as determined by one-way ANOVA (*p* ≤ 0.05).

*S. rhizophila* Ep2.2 developed on NGA medium as a pale-yellow glistening bacterial colony, with entire margins. The pure isolate was Gram negative, catalase and oxidase positive, and unable to grow at 4°C and 37°C. The strain showed proteolytic, lipolytic and chitinolytic activities but it did not produce β-glucanases (**Table 1**). *S. rhizophila* Ep2.2 was also unable to produce siderophores, extracellular polymeric substances (EPS), indole-3-acetic acid (IAA), ACC deaminase, and to solubilize phosphate (**Table 1**). *S. rhizophila* Ep2.2 displayed a marked ability to form biofilm, similarly to *S. maltophilia* LMG 958, as well as significant swimming and swarming abilities which were comparable, although statistically different, to those of other tested bacterial species (**Figure 2**). In particular, *S. rhizophila* Ep2.2 showed higher swimming and swarming abilities, and lower twitching ability than *S. maltophilia* LMG 958 (**Figure 2**).

**Table 1:** Biochemical and physiological characteristics of *Stenotrophomonas rhizophila* strain Ep2.2 and *Stenotrophomonas malthophilia* LMG 958 reference strain.

	*S. rhizophila* Ep2.2	*S. maltophilia* LMG 958
Gram reaction	–	–
Catalase	+	+
Oxidase	+	+
Growth^a^ at 4°C	–	–
Growth^a^ at 30°C	+	+
Growth^a^ at 37°C	–	+
Growth^a^ at 40°C	–	+
Protease	+	+
Lipase	+	+
β-glucanase	–	–
Chitinase	+	+
Siderophore	–	–
Biofilm formation^b^	+	+
EPS	–	–
IAA	–	nt
ACC deaminase	–	nt
Phosphate solubilization	–	nt

+, tested positive; -, tested negative; nt, not tested.

a Growth at different temperatures was determined in LB medium.

b Values of absorbance OD_600nm_ measured for the different species were: *S. rhizophila* = 1.89; *S. maltophilia* = 1.76; *A. tumefaciens* = 1.12; *X. campestris* pv. *campestris* = 0.96.

**Figure 3:**
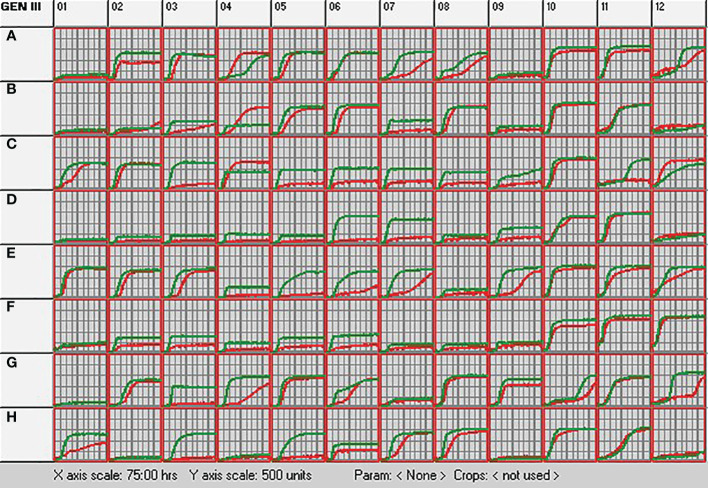
Metabolic profiling of *S. rhizophila* Ep2.2 (red curve) and *S. malthophilia* LMG 958 (green curve) as resulting by the colorimetric curves produced with the OmniLog device. The two species were grown in GEN III MicroPlates (BIOLOG) for 75 hours at 30°C. Measurements were performed every 15 min. The curves indicate utilization of the carbon sources or resistance to the inhibitory chemicals. Columns 1-9, carbon source utilization assays; Columns 10-12, chemical sensitivity assays. Details on the microplate content are shown in **Supplementary Figure S5** and commented in the Results section 3.1.

The metabolic profile of *S. rhizophila* Ep2.2 was unambiguously different from that of *S. maltophilia* LMG 958 (**Figure 3**). In general, the metabolic response of *S. rhizophila* Ep2.2 was slower or reduced in comparison to *S. maltophilia* LMG 958, except for D-trehalose, β-Methyl-D-Glucoside, and D-galactose on which *S. maltophilia* Ep2.2 displayed a better utilization capacity (**Figure 3**: wells A4, B4, and C4, respectively). However, unlike *S. maltophilia* LMG 958, *S. rhizophila* Ep2.2 either did not or barely utilize N-acetyl-β-D-mannosamine, D-fructose, 3-methyl glucose, D-fucose, L-fucose, L-rhamnose, inosine, D-glucose-6-phosphate, D-fructose-6-phosphate, D-serine, L-arginine, L-aspartic acid, L-glutamic acid, D-galacturonic acid, L-galactonic acid lactone, D-gluconic acid, D-glucuronic acid, glucuronamide, D-lactic acid methyl ester, α-Hydroxy-Butyric Acid, and α-Keto-Butyric Acid (**Figure 3**: wells B7, C3, C5, C6, C7, C8, C9, D6, D7, D9, E4, E5, E6, F2, F3, F4, F5, F6, G3, H3, and H5, respectively). *S. rhizophila* Ep2.2 resulted more sensitive to fusidic acid and potassium tellurite (**Figure 3**: wells C11 and G12). Noteworthy, the sensitivity to NaCl, previously known to discriminate *S. rhizophila* from *S. maltophilia* (Wolf et al., 2002), was similar between the two *Stenotrophomonas* strains tested here (i.e. tolerance to 1 and 4% NaCl; sensitivity to 8% NaCl) (**Figure 3**: wells B10-12). Moreover, both *S. rhizophila* and *S. maltophilia* strains showed sensitivity to minocycline (**Figure 3**: well D12). Distinct metabolic curves were observed concerning growth at pH 5, and regarding utilization of dextrin, sucrose, D-turanose, L-histidine, L-serine, L-lactic acid and Tween 40 (**Figure 3**: wells A12, A2, A7, A8, E7, E9, G4, and H1).

### 3.2 *Stenotrophomonas rhizophila* Ep2.2 inhibits growth of fungal plant pathogens *in vitro*

The inhibitory activity of *S. rhizophila* Ep2.2 was assessed against four fungal plant pathogens able to infect tomato plants: *B. cinerea*, *A. alternata*, *F. oxysporum* f. sp. *lycopersici* and *R. solani*. Two different methods were used: dual culture assay, where both *S. rhizophila* Ep2.2 and the pathogenic fungus were grown on the same PDA medium (in a Petri dish not sealed with parafilm), and a two-compartment assay where *S. rhizophila* Ep2.2 and the pathogenic fungus were grown physically separated, each one on its appropriate medium (NGA and PDA, respectively), while the headspace containing volatile compounds was shared. The two-compartment Petri dish was sealed with parafilm.

**Figure 4:**
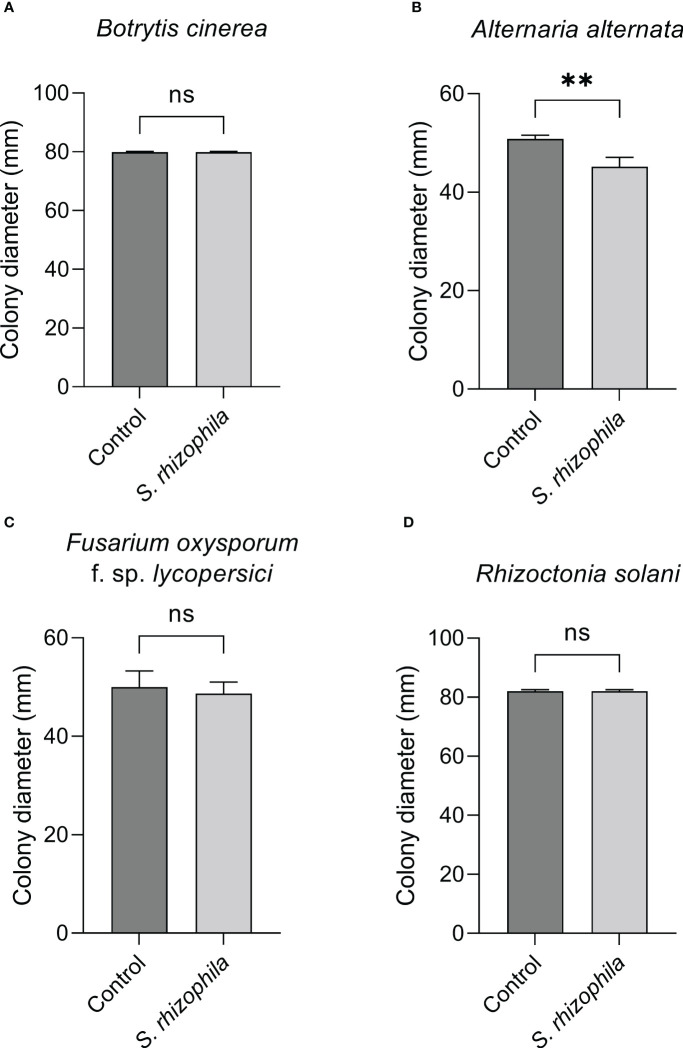
Dual culture assays against fungal tomato pathogens. *Botrytis cinerea* **(A)**, *Alternaria alternata* **(B)**, *Fusarium oxysporum* f. sp. *lycopersici* **(C)** and *Rhizoctonia solani* **(D)** were grown in the presence of *S. rhizophila* Ep2.2 in Petri dishes not sealed with parafilm. Colony diameters were measured after 6 days of growth at 26°C. Values are reported as mean ± SD, *n* = 3. The experiment with *B. cinerea* was repeated with similar results. Asterisks indicate statistically significant differences at *p* < 0.01 (**); ns, not significant.

The dual culture assay highlighted a mild inhibitory activity of *S. rhizophila* Ep2.2 against *A. alternata* (~11% growth reduction), whereas *B. cinerea*, *R. solani* and *F. oxysporum* f. sp. *lycopersici* were not significantly affected by the presence of *S. rhizophila* Ep2.2 after 6 days of growth on PDA medium (**Figure 4**).

**Figure 5:**
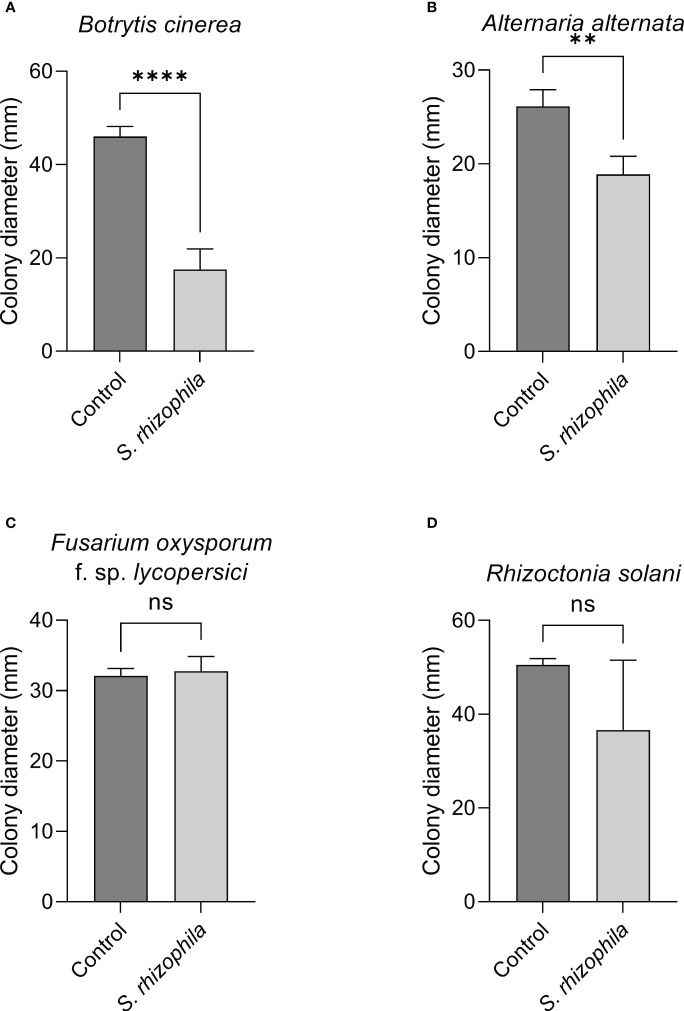
Effect of volatile compounds from *S. rhizophila* Ep2.2 on the growth of fungal tomato pathogens *in vitro*. *Botrytis cinerea* **(A)**, *Alternaria alternata* **(B)**, *Fusarium oxysporum* f. sp. *lycopersici* **(C)** and *Rhizoctonia solani* **(D)**. The assay was conducted in two-compartment Petri dishes sealed with parafilm. Colony diameters were measured after 3 days of growth at 26°C. Values are reported as mean ± SD, *n* = 4. The test with *B. cinerea* was repeated and similar results were obtained. Asterisks indicate statistically significant differences at *p* < 0.01 (**) or *p* < 0.0001 (****); ns, not significant. Representative pictures are included as **Supplementary Figure S2**.

In contrast, when the two-compartment assay was performed, the growth of both *B. cinerea* and *A. alternata* was markedly inhibited by *S. rhizophila* Ep2.2 (~62% and ~28% growth reduction, respectively), whereas no significant reduction was detectable for *R. solani* and *F. oxysporum* f. sp. *lycopersici* (**Figure 5**). These results highlighted a role for volatile compounds in inhibiting the growth of microbial pathogens, especially *B. cinerea* (**Figures 4**, **5** and **Supplementary Figure S2**).

**Figure 6:**
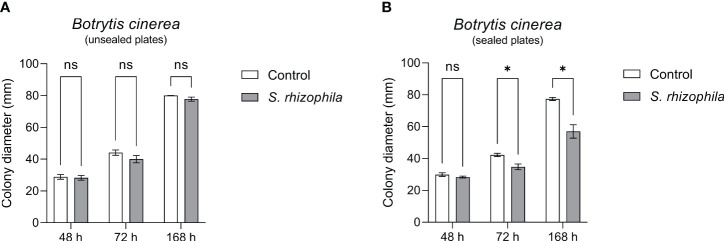
Dual culture assays against *Botrytis cinerea* in Petri dishes unsealed **(A)** and sealed **(B)** with parafilm. Colony diameters were measured after 48, 72 and 168 hours of growth at 26°C. Values are reported as mean ± SEM, *n* = 4. Asterisks indicate statistically significant differences at *p* ≤ 0.05 (*); ns, not significant.

In order to confirm this clue, the dual culture assay between *S. rhizophila* Ep2.2 and *B. cinerea* was repeated by sealing the plates with parafilm, and *B. cinerea* growth was measured after 48, 72 and 168 h by comparing sealed with unsealed plates. As shown in **Figure 6**, whereas *B. cinerea* was not significantly affected by *S. rhizophila* Ep2.2 in unsealed plates, its growth was significantly inhibited in parafilm-sealed plates (**Figure 6**). In addition, when a culture filtrate from *S. rhizophila* Ep2.2 was produced and tested again *B. cinerea* no significant reduction in growth was observed as compared to control (**Supplementary Figure S1**). This result excluded an inhibitory role of diffusible molecules released by the bacterium into the medium. Overall, these results suggested the production by *S. rhizophila* Ep.2.2 of volatile compounds able to inhibit the growth of fungal plant pathogens (*A. alternata* and *B. cinerea*) and *B. cinerea* was exclusively inhibited by these compounds.

### 3.3 Headspace analysis of VOCs emitted *in vitro* by *Stenotrophomonas rhizophila* Ep2.2

Real-time screening by PTR-MS of VOCs present in the headspace of axenic cultures of *S. rhizophila* revealed a complex blend, although 10 protonated ions represented 97.9% of the total (**Table 2** and **Supplementary Table S2**). In particular, three protonated ions resulted to be mainly present: *m/z* = 33 (32.3 ± 4.9%), which was unambiguously assigned to methanol, followed by *m/z* = 97 (30.1 ± 43.3%) and *m/z* = 49 (21.8 ± 13.8%). Because of the high relative humidity of the headspace, the abundant presence of methanol generated a small percentage of a water-clustered methanol (i.e. methanol-H_2_O) detectable at *m/z* = 51 (2.0 ± 15.7%). Simultaneous GC-MS analysis of the same samples (**Supplementary Table S3**) allowed identifying the protonated ion *m/z* = 97 as 2, 4-dimethyl furan, whereas *m/z* = 49 was assigned to methanethiol (**Table 2**). Among the VOCs emitted in higher percentage, other sulphur containing compounds such as dimethyl sulfide (DMS) and dimethyl disulfide (DMDS) were detected by PTR-MS at *m/z* = 63 and at *m/z* = 95, respectively, as confirmed by GC-MS identification (**Table 2** and **Supplementary Table S3**). Moreover, the PTR-MS analysis detected a protonated ion at *m/z* 99 (4.0 ± 52.2%) that we assigned to a fragment of hexanoic acid rather than to hexenals, based on previous analyses (Bergamaschi et al., 2015) and because we did not detect the fragment at *m/z* 81, which is typically produced from the fragmentation of hexenals following proton transfer reaction (Brilli et al., 2011). We assigned to hexanals the protonated ion at *m/z* 101 due to the concomitant presence of *m/z* 83 (**Supplementary Table S3**), which is its main fragment (Brilli et al., 2011).

**Table 2:** The 10 most abundant protonated ions related to VOCs and/or fragments of VOCs detected by PTR-QMS from axenic cultures of *Stenotrophomonas rhizophila* Ep2.2, which represent ~ 98% of the overall blend of VOCs.

Protonated ion (*m/z*)	Relative amount within the VOC blend (%) ± error (%)	Assignment to specific VOCs/fragment of VOCs
33	32.3 ± 4.9	methanol
97	30.9 ± 43.3	2,4-dimethyl furan*
49	21.8 ± 4.0	methanethiol*
99	4.0 ± 52.2	e.g. hexanoic acid fragment
43	2.7 ± 442.3	hexanal fragment
51	2.0 ± 15.7	methanol-water cluster
98	1.7 ± 54.5	n.a.
63	1.5 ± 30.6	dimethyl sulfide (DMS)*
95	0.6 ± 278.5	dimethyl disulfide (DMDS)*
101	0.4 ± 125.3	hexanals

The remaining ions are shown in **Supplementary Table S2**. Mean values were calculated from 7-9 different bacterial cultures from two independent experiments and indicate the percentage of protonated ions related to VOCs and/or fragment of VOCs on the total protonated ions detected, whereas ± errors express, in percentage, the standard error of the different replicates with respect to their raw mean values. (*) indicates VOCs which have been further identified by GC-MS analysis; n.a., not assigned.

**Figure 7:**
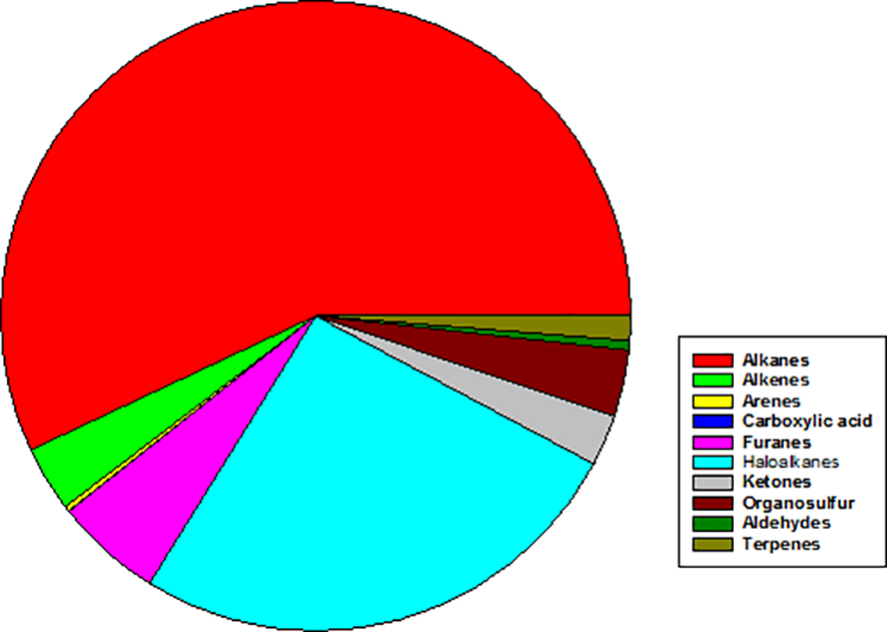
Mean abundance of different chemical classes of VOCs emitted by axenic cultures of *Stenotrophomonas rhizophila* Ep2.2 as identified by GC-MS analysis. In particular, % values are: Alkanes = 56.97%; Alkenes = 3.36%; Arenes = 0.27%; Carboxylic acid = 0.03%; Furans = 5.49%; Haloalkanes = 26.04%; Ketones = 2.62%; Organosulfur compounds = 3.42%; Aldehydes = 1.26%; Terpenes = 0.02%.

Complementary analysis by GC-MS, which has different sensitivity and selectivity than PTR-MS, highlighted the capacity of *S. rhizophila* to emit a wide variety of VOCs belonging to different chemical classes (**Figure 7**). Among those, the most abundant VOCs resulted to be the haloalkane trichloromethane (26.0 ± 15.7%), the alkanes 2,4-dimethyl heptane (9.5 ± 8.8%) and 4-methyl octane (8.0 ± 9.1%), followed by furans (furan = 2.4 ± 8.4% and 2,4-dimethyl furan = 3.0 ± 31.2%) and the organosulfur compound dimethyl sulfide (3.2 ± 8.9%). Terpenes, such as α- and β-pinene, camphene, and Δ-3-carene were present in very small percentage (< 0.1%) (**Supplementary Table S3**).

### 3.4 *Stenotrophomonas rhizophila* Ep2.2 restricts *Botrytis cinerea* infection and primes defense genes in tomato leaves

**Figure 8:**
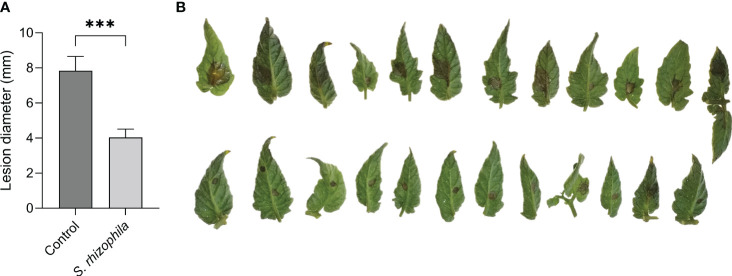
*S. rhizophila* Ep2.2 protects tomato leaves from *B. cinerea* infection. Tomato cv. Micro-Tom leaves were treated with a bacterial suspension of *S. rhizophila* Ep2.2 and inoculated 48 hours later with *B. cinerea* (10-µL drops of 1×10^6^ conidia/mL). Lesions caused by *B. cinerea* were measured after 3 days of incubation (mean ± SEM, *n* = 12-13) **(A)**. Asterisks indicate statistically significant differences at *p* < 0.001 (***). Pictures were taken on the same day **(B)**; upper line, control leaves infected with *B. cinerea*; lower line, leaves treated with *S. rhizophila* Ep2.2 and infected with *B. cinerea*. The experiment was performed three times with similar results.

To assess the ability of *S. rhizophila* Ep2.2 to protect plant tissues against *B. cinerea* infection, leaves from tomato cv. Micro-Tom were detached, sprayed with *S. rhizophila* Ep2.2, incubated for 48 h under high RH conditions, and subsequently inoculated with *B. cinerea* conidia. As shown in **Figure 8**, the treatment with *S. rhizophila* Ep2.2 strongly reduced *B. cinerea* colonization of leaf tissues. The disease severity, as determined by the lesion size diameters, was significantly reduced after three days of incubation (~50%). After four days, the lesion size reduction was even greater (**Supplementary Figure S3**). The protective effect induced by *S. rhizophila* Ep2.2 against *B. cinerea* was clearly reproducible on whole tomato plants of a different cultivar (Marmande) (**Supplementary Figure S4**).

A gene expression analysis was performed to investigate the contribution of induced resistance to the protective effect shown by *S. rhizophila* Ep2.2 against *B. cinerea* infection in tomato leaves. This analysis was designed to highlight either the local induction of plant defense genes before infection, or their quicker or stronger expression upon infection (i.e. defense priming). Genes were selected based on their involvement in defense responses to *B. cinerea* as reported in Baccelli et al. (2022). Before RNA extraction, the outcome of *B. cinerea* infection was verified to make sure that leaf samples were displaying increased protection against *B. cinerea* infection (**Supplementary Figure S3**).

**Figure 9:**
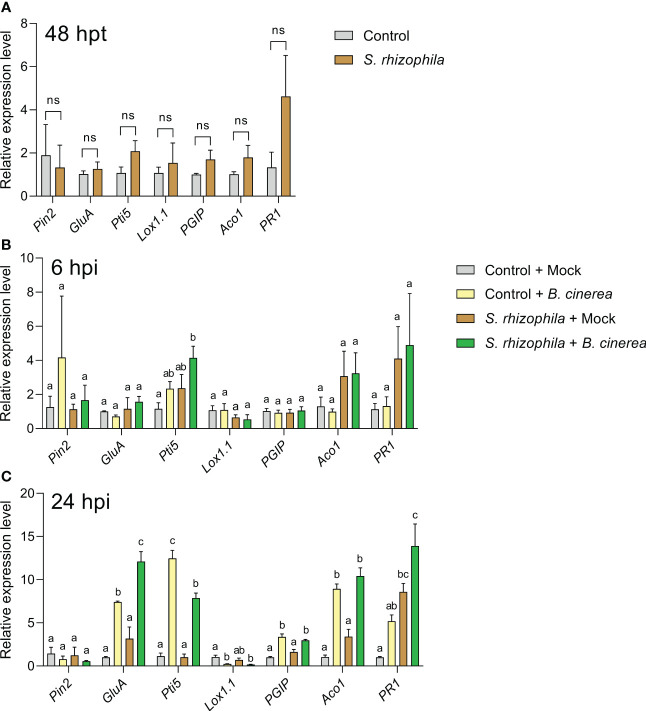
Expression of defense genes in *Solanum lycopersicum* cv. Micro-Tom leaves sprayed with *S. rhizophila* Ep2.2 **(A)** and subsequently inoculated with *B. cinerea* conidia **(B, C)**. Mock infections were performed with potato dextrose broth (PDB). hpt, hours post treatment; hpi, hours post infection. Genes analyzed by RT-qPCR: *PIN2 (Proteinase inhibitor II)*, *GluA* (*β-1,3-glucanase* **(A)**) *PTI5 (Pathogenesis-related genes transcriptional activator 5), Lox1.1 (Lipoxygenase A), PGIP (Polygalacturonase inhibitor protein), ACO1* (*1-aminocyclopropane-1-carboxylate oxidase 1*), *PR1 (Pathogenesis-related protein 1). Actin-7* was used as the endogenous reference gene for transcript normalization. Control [in **(A)**] or control + mock at 6 or 24 hpi [in **(B, C)** respectively] samples were used as calibrators (grey bar) for relative gene expression calculations. Mean fold change values ± SEM are shown (*n* = 3). Statistical analysis per each gene at each specific time point was performed by one-way ANOVA. Significant differences are marked by different letters (*p* ≤ 0.05); ns, not significant.

After 48 h following treatment with *S. rhizophila*, and prior to *B. cinerea* inoculation, no significant changes in the expression of defense genes were detectable in tomato leaves compared to control (**Figure 9A**). In contrast, 6 h after *B. cinerea* inoculation (**Figure 9B**), a significant up-regulation of the *Pti5* gene was detectable only in tomato leaves previously treated with *S. rhizophila* (“*S. rhizophila* + *B. cinerea*” samples) (**Figure 9B**), thus indicating a quick response to pathogen infection in *S. rhizophila*-treated leaves. After 24 h, all the genes investigated were significantly modulated by the infection (24 hpi) with the exclusion of *Pin2*, whose levels remained unaltered (**Figure 9C**). The *GluA* gene was significantly up-regulated in *B. cinerea*-infected tomato leaves regardless of *S. rhizophila* treatment, although in *S. rhizophila-*treated leaves the expression level was significantly higher (**Figure 9C**), indicating a stronger response to pathogen infection in leaves pre-treated with *S. rhizophila*. In contrast, the *Pti5* gene was up-regulated to a lower extent by *B. cinerea* infection in the leaves pre-treated with *S. rhizophila* (**Figure 9C**). The *Lox1.1* gene was strongly downregulated by the infection with *B. cinerea*, whereas *PGIP* and *Aco1* genes were significantly upregulated, irrespective of the treatment with *S. rhizophila*. After 24 h, the *PR1* gene was significantly up-regulated only in the leaves pre-treated with *S. rhizophila* (i.e. “*S. rhizophila* + Mock” and “*S. rhizophila* + *B. cinerea”*) (**Figure 9C**), whereas no significant up-regulation was detectable in *B. cinerea* infected leaves which were not treated with *S. rhizophila* (“control + *B. cinerea”*), indicating that the beneficial bacterium was able to enhance *PR1* gene expression.

Overall, the gene expression analyses suggested that inoculation with *S. rhizophila* did not cause major changes in gene expression in healthy tomato leaves, with the exception of *PR1* gene up-regulation at late time points (24 hpi, i.e. 72 h after *S. rhizophila* inoculation on leaves, **Figure 9C**). However, the treatment with *S. rhizophila* led to the early up-regulation of the *Pti5* gene (6 hpi) and to the enhanced up-regulation of *GluA* and *PR1* genes (24 hpi) during infection with *B. cinerea*.

## 4 Discussion

With the present work, we provide a thorough biochemical and metabolic characterization of a novel phyllosphere epiphytic strain of *S. rhizophila* and show how this bacterium can protect tomato leaves from infection by *B. cinerea*, a polyphagous pathogenic fungus able to infect hundreds of plant species on their above-ground organs (Dean et al., 2012).

A well-known distinctive feature of *S. rhizophila* is the inability to grow at the human body temperature, a trait that has been explained with the lack of heat-shock genes and the probable activation of suicide mechanisms occurring at high temperatures (Alavi et al., 2014). In accordance with this evidence, the *S. rhizophila* strain Ep2.2 that we isolated with this work was unable to grow at 37°C or 40°C, unlike we observed for *S. maltophilia* LMG 958.

*S. rhizophila* Ep2.2 showed higher swimming and swarming abilities than *S. maltophilia* LMG 958, but lower twitching ability. Bacterial swimming and swarming motilities are powered by rotating flagella, whereas twitching is powered by the extension and retraction of type IV pili (Kearns, 2010). Motility in plant-associated bacteria is important for survival and host colonization (Turnbull et al., 2001). A comparative genomic analysis carried out to detect genes and functions useful to differentiate plant beneficial and human pathogenic *Stenotrophomonas* strains evidenced several genes responsible for motility in *S. rhizophila* (Alavi et al., 2014), supporting both rhizosphere and phylloplane competency. It is reasonable to hypothesize here that the marked swimming and swarming abilities displayed by *S. rhizophila* Ep2.2 can be related to its epiphytic lifestyle.

Our metabolic profiling of *S. rhizophila* Ep2.2 provided new insights on the chemical sensitivity and capability of this bacterium to use various carbon sources. The sensitivity to NaCl, previously reported to be lower for *S. rhizophila* (Wolf et al., 2002), was instead similar between *S. rhizophila* Ep2.2 and *S. malthophilia* LMG 958: both strains were able to tolerate 1% and 4% NaCl, while they were negatively affected by 8% NaCl. In addition, both strains displayed sensitivity to minocycline, a tetracycline antibiotic used to treat *S. malthophilia* infections in humans (Hand et al., 2016). In contrast, *S. rhizophila* Ep2.2 was more sensitive to fusidic acid and potassium tellurite than *S. malthophilia* LMG 958. It is worth nothing here that our results confirm the high tolerance of *S. malthophilia* to tellurite (Pages et al., 2008).

In the carbon source utilization assays, *S. rhizophila* Ep2.2 generally showed a slower response to several tests and a reduced utilization ability as compared to *S. malthophilia* LMG 958. However, *S. rhizophila* Ep2.2 showed better utilization capacity for D-trehalose, β-Methyl-D-Glucoside and D-galactose. The result of trehalose utilization is noteworthy, since a previous genomic comparison between the two species pointed to the *ThuA* gene, encoding an enzyme involved in trehalose utilization, as characteristic of the species *S. rhizophila*, being absent in *S. malthophilia* (Pinski et al., 2020). Therefore, we may assume that the *ThuA* gene is responsible for the difference in D-trehalose utilization that we observed.

Overall, the biochemical and metabolic data confirmed the genetic identification of *S. rhizophila* Ep2.2 while revealed differences and similarities between *S. rhizophila* and *S. maltophilia* never been reported so far.

In this work, we also analyzed the potential of *S. rhizophila* Ep2.2 to protect tomato plants from pathogens. To do so, we first performed analyses *in vitro* by focusing on four fungal pathogens. The antifungal activity of the species *S. rhizophila* has been known since its first identification (Wolf et al., 2002), and several studies have suggested the involvement of VOCs (Kai et al., 2007; Cernava et al., 2015; Reyes-Perez et al., 2019; Rivas-Garcia et al., 2019). Here, we clearly demonstrate that VOCs produced by *S. rhizophila* Ep2.2 not only contribute to the antifungal activity against *A. alternata*, but are the only determinants of the activity against *B. cinerea in vitro*. This is consistent with Rojas-Solís et al. (2018), whose study led to a similar conclusion concerning the antifungal activity of *S. maltophilia* against *B. cinerea*.

In this regard, we thoroughly screened VOC emissions from *S. rhizophila* Ep2.2 by combining PTR-MS and GC-MS analyses. The capability of PTR-MS to detect a wide range of VOCs in real-time was complemented by that of GC-MS. This because while PTR-MS enables the analysis of VOCs by avoiding preselection bias, it cannot distinguish VOCs and/or fragments of VOCs having the same molecular weight. On the other hand, the selectivity of both the sampling adsorbent materials and the gas chromatography column limits the variety of VOCs to be analyzed, especially those having a low molecular weight (for instance methanol), although recognition by mass spectrometry allows identification of ambiguous protonated ions related to VOCs (Sharifi et al., 2022). In accordance with Shestivska et al. (2015), our PTR-MS analysis confirmed methanol to be among the most abundant VOCs within the blend produced by *S. rhizophila* Ep2.2. Bacteria commonly emit methanol as a product mainly resulting from metabolic processes of demethoxylation of cellular polysaccharides (Mincer and Aicher, 2016; Misztal et al., 2018). Likewise, abundant emission of alkanes and haloalkanes detected by GC-MS could be a general feature of *S. rhizophila* Ep2.2 metabolism (Ladygina et al., 2006; Klähn et al., 2014; Weigold et al., 2016). Among the main protonated ions detected by PTR-MS within the VOC blend, 2,4-dimethyl furan was found in the highest percentage, and it was unambiguously identified by GC-MS analysis. The antifungal activity of dimethyl furan emitted by bacteria has been recently demonstrated against various fungal plant pathogens (Lin et al., 2021). Moreover, both PTR-MS and GC-MS analyses confirmed the emission of DMS and DMDS by *S. rhizophila* Ep2.2. We assigned to methanethiol the protonated ion *m/z* 49, which was recorded by PTR-MS as one of the main constituents of the VOC blend, although it was not detected by the GC-MS analysis. Since methanethiol had been detected by the same GC-MS system in a previous investigation as a highly abundant VOC emitted by *Pseudomonas chlororaphis* (Raio et al., 2020), we believe that the low sensitivity of our GC column for sulphur compounds may have limited its detection in the present study case. All these sulphur-containing VOCs emitted by *S. rhizophila* Ep2.2 may show toxicity against plant pathogens due to the bonding of S-functional groups to reactive sites in fungi (Baerlocher et al., 1999; Groenhagen et al., 2013). In particular, DMS has been demonstrated to have antifungal activity both *in vitro* (Wang et al., 2013) and *in vivo* when fumigated to plants (Li et al., 2010). We also detected, by both PTR-MS and GC-MS, trace emissions of terpenes (i.e. monoterpenes) from *S. rhizophila* Ep2.2. Terpenes play biological and ecological roles in bacteria to cope with different (a)biotic stresses, and may act as infochemicals in mediating microbial interactions (Avalos et al., 2022). Bacterial terpenes (i.e. β-pinene) can also show inhibitory activity against fungi (Song et al., 2015). Our PTR-MS analysis also detected, among the main VOCs emitted by *S. rhizophila* Ep2.2, a small percentage of hexanals which possess antifungal activity (Zhang et al., 2021). Therefore, the synergy of furans, sulphur containing VOCs and terpenes can explain the high antifungal activity shown by *S. rhizophila* Ep2.2 against *A. alternata* and *B. cinerea in vitro*.

The novel information we provide on the blend of VOCs emitted by *S. rhizophila* highlights a species-specific inhibitory activity of *S. rhizophila* against various pathogenic fungi (Kai et al., 2007). The blend of VOCs we detected can be the result of specific peculiarities of the *S. rhizophila* Ep2.2 strain (Lo Cantore et al., 2015), as well as be influenced by the growth medium and conditions used in this study (Blom et al., 2011). In fact, with respect to the study of Shestivska et al. (2015), we cultured *S. rhizophila* on nutrient glucose agar (NGA) medium rather than Mueller-Hinton broth (MHB) liquid medium, incubated at 26°C rather than 30°C, and for a longer period of time. However, our analytical instrumentation and assay contribute to the complete characterization of the VOC profile emitted by *S. rhizophila*, as we employed both a different absorbent material and GC-column type than that of Shestivska et al. (2015), as well as a PTR-MS rather than a selected ion flow tube mass spectrometry (SIFT-MS) having a different sensitivity for VOCs, in addition to avoid problems related to the use of solvents (Kai et al., 2007).

When sprayed on tomato leaves and plants, *S. rhizophila* Ep2.2 was able to restrict *B. cinerea* colonization. Beneficial microbes are known to stimulate the plant’s immune system for enhanced defense responses to pathogen infection (Pieterse et al., 2014; Syed Ab Rahman et al., 2018). Induced resistance in plants may involve both direct elicitation of defenses ahead of infection and their quicker/stronger activation upon infection. This latter phenomenon is termed “defense priming” (Mauch-Mani et al., 2017). Beneficial bacteria may produce and release various molecules with resistance-inducing/priming activity (Pieterse et al., 2014; Mauch-Mani et al., 2017). Our PTR-MS analysis indicated for instance the production of hexanoic acid, a well-known priming compound able to enhance resistance in *B. cinerea*-infected tomato plants (Aranega-Bou et al., 2014).

The detached leaf assay used here appears to be a reliable system to reveal both gene expression changes and resistance induction. Control leaves, in fact, responded to *B. cinerea* at the gene expression level in a similar manner to what previously observed on plants and were also similarly susceptible to infection (Baccelli et al., 2022). The gene expression analyses demonstrate that leaf inoculation with *S. rhizophila* Ep2.2 induces resistance in leaves. In particular, while defense genes were not pre-activated by *S. rhizophila* before *B. cinerea* infection, these were up-regulated either more quickly (*Pti5* gene) or to a higher extent (*GluA* and *PR1* genes) during *B. cinerea* infection. This suggests that leaves were primed by *S. rhizophila* for an enhanced pathogen defense (Martinez-Medina et al., 2016).

*B. cinerea* is a necrotrophic plant pathogen able to enter the host trough stomata or by penetrating directly the cuticle (Bi et al., 2023). In the early phases of infection, *B. cinerea* produces molecules able to promote plant cell death; subsequently (24-48 hpi), the fungus has to defend itself from the attack of plant antimicrobial compounds to spread further into the plant tissues (Bi et al., 2023). The genes analyzed in this study are all known to be involved in defense signaling or encode antifungal enzymes in tomato (Baccelli et al., 2022). *PR1* expression is considered as a marker of salicylic acid (SA)-dependent defenses and has been also associated to priming to both necrotrophic and biotrophic pathogens (Mauch-Mani et al., 2017). The *Pti5* gene, which was primed 6 h following infection with *B. cinerea* in our experiment, encodes a transcription factor that has been reported to accelerate pathogen-induced expression of defense-related genes, among those the *Glucanase B* and *PR1* genes (He et al., 2001; Wang et al., 2021). In *Arabidopsis*, the expression of *Pti5* gene from tomato has been reported to activate the SA-regulated genes *PR1* and *PR2* (β-1,3-glucanase) (Gu et al., 2002). Noteworthy, our results actually showed enhanced transcription of *β-1,3-glucanase A (GluA)* and *PR1* genes 24 h after *B. cinerea* infection, allowing to hypothesize a link between the early expression of *Pti5* and the subsequent enhanced expression of the SA-associated genes *GluA* and *PR1* in *S. rhizophila* primed plants. This picture is consistent with former studies that indicate a role for SA-dependent defenses in restricting *B. cinerea* infection in tomato and *Arabidopsis* (Zimmerli et al., 2001; Achuo et al., 2004).

The resistance-inducing ability of *Stenotrophomonas* spp. in plants has been scarcely studied so far. Root colonization with *S. maltophilia* SBP-9 was found to increase resistance to *F. graminearum* infection in wheat plants by enhancing the activity of antioxidant enzymes and β-1,3-glucanases (Singh and Jha, 2017), suggesting the induction of priming. More recently, soil inoculation with *S. rhizophila* SR80 was reported to increase resistance to *F. pseudograminearum* in wheat by boosting the expression of defense-related genes during pathogen infection (Liu et al., 2021). Our results clearly show that tomato leaves inoculated with *S. rhizophila* were more protected from *B. cinerea* infection, and this was likely due to a primed state that allowed quicker and stronger expression of defense genes. Despite we cannot exclude that the emission of antifungal VOCs by *S. rhizophila* may have contributed to hinder leaf colonization by *B. cinerea*, the occurrence of defense priming at the gene expression level was demonstrated, and it is tempting to speculate that some VOCs produced by *S. rhizophila* may act as priming-inducing stimuli.

## 5 Conclusion

To safeguard yields, we need to protect our crops from stresses and diseases. New eco-friendly approaches are currently strongly demanded for this purpose, such as those employing beneficial microbes that crops may have lost either during domestication or because of intensive agricultural practices. With this study, we highlight the potential of a phyllosphere epiphytic strain of *S. rhizophila* isolated from an ornamental plant as foliar inoculant to control leaf infection in crops, specifically in tomato. Our results reveal the production of antifungal VOCs by *S. rhizophila* Ep2.2 and a priming effect at the gene expression level that may contribute to inhibit pathogen growth and host colonization.

## Data availability statement

The original contributions presented in the study are included in the article/**Supplementary Material**. Further inquiries can be directed to the corresponding author. The 16S rDNA sequence of the strain Ep2.2 was deposited in NCBI, accession number MZ841807.

## Author contributions

AR and IB conceived and designed the present study. IB and XC conceived the main project. AR, FB, LN, RB, FO, CP and IB carried out the experiments and collected data. IB and FB analyzed data and prepared figures and tables. AR, FB and IB wrote the initial draft. All authors provided their feedbacks during manuscript preparation. All authors contributed to the article and approved the submitted version.
