# Supplementary material for: Stenotrophomonas rhizophila Ep2.2 inhibits growth of Botrytis cinerea through the emission of volatile organic compounds, restricts leaf infection and primes defense genes
Source: Front Plant Sci. 2023 Oct 2;14:1235669. doi: 10.3389/fpls.2023.1235669 (PMC10577304; doi:10.3389/fpls.2023.1235669)
Supplement: Supplementary file 1 [file DataSheet_1.docx]

Supplementary Material

***Stenotrophomonas rhizophila* Ep2.2** **inhibits growth of *Botrytis cinerea* through the emission of volatile organic compounds, restricts leaf infection and primes defense genes**

**Aida Raio, Federico Brilli, Luisa Neri, Rita Baraldi, Francesca Orlando, Claudio Pugliesi, Xiaoyulong Chen, Ivan Baccelli***

*** Correspondence:** Corresponding Author, Email: [ivan.baccelli@ipsp.cnr.it](mailto:ivan.baccelli@ipsp.cnr.it)

**Supplementary Figure S1.** *Botrytis cinerea* grown on PDA medium amended with a culture filtrate of *Stenotrophomonas rhizophila* Ep2.2. Colony diameters were measured after 4 days of growth at 26°C. Mean values ± SD are reported (control, *n* = 3; culture filtrate, *n* = 4); ns, not significant.


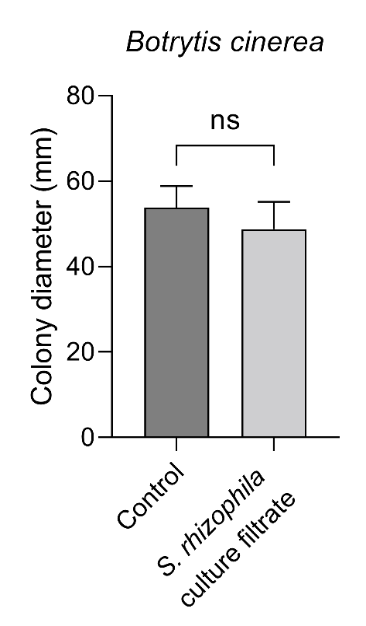


**Supplementary Figure S2.** Representative pictures showing the effect of volatile compounds emitted by *S. rhizophila* Ep2.2 on the growth of *Botrytis cinerea* **(A, B)** or *Alternaria alternata* **(C, D)**. *S. rhizophila* Ep2.2 is present on the left side of the Petri dishes in **B** and **D**. Pictures were taken after 3 days of growth at 26°C.

**
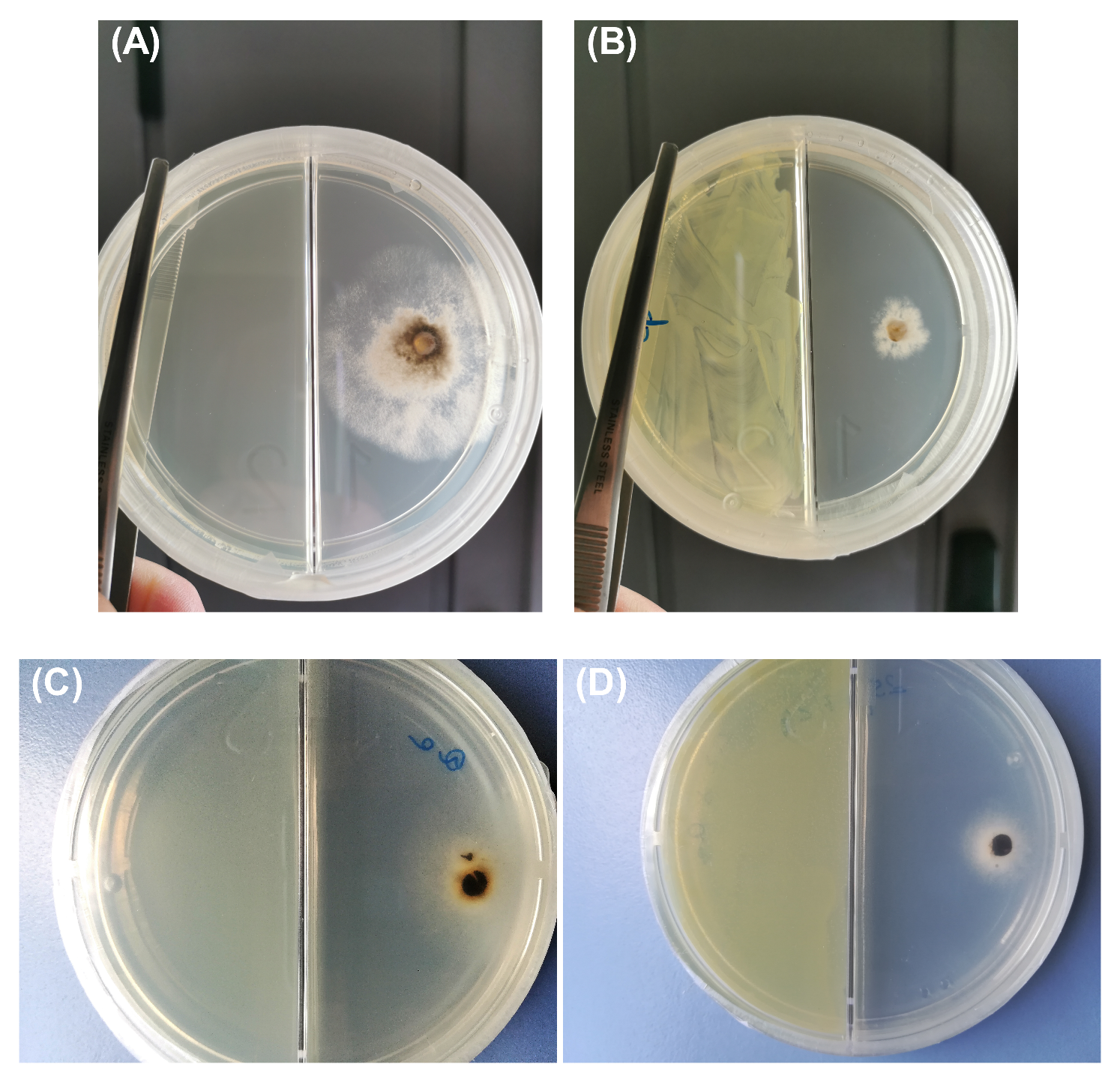
**

**Supplementary Figure S3**. Infection development on leaves not sampled for RNA extraction. Tomato cv. Micro-Tom leaves were sprayed with a bacterial suspension of *S. rhizophila* Ep2.2 and inoculated 48 h later with *B. cinerea* conidia. Necrotic lesions caused by *B. cinerea* were measured after 4 days of incubation (**A**). Values are shown as mean ± SEM (*n* = 10). Asterisks indicate statistically significant differences at *p* < 0.0001 (****). Representative pictures (**B**) were taken on the same day: upper line, control leaves infected with *B. cinerea*; lower line, leaves treated with *S. rhizophila* Ep2.2 and infected with *B. cinerea*.

**
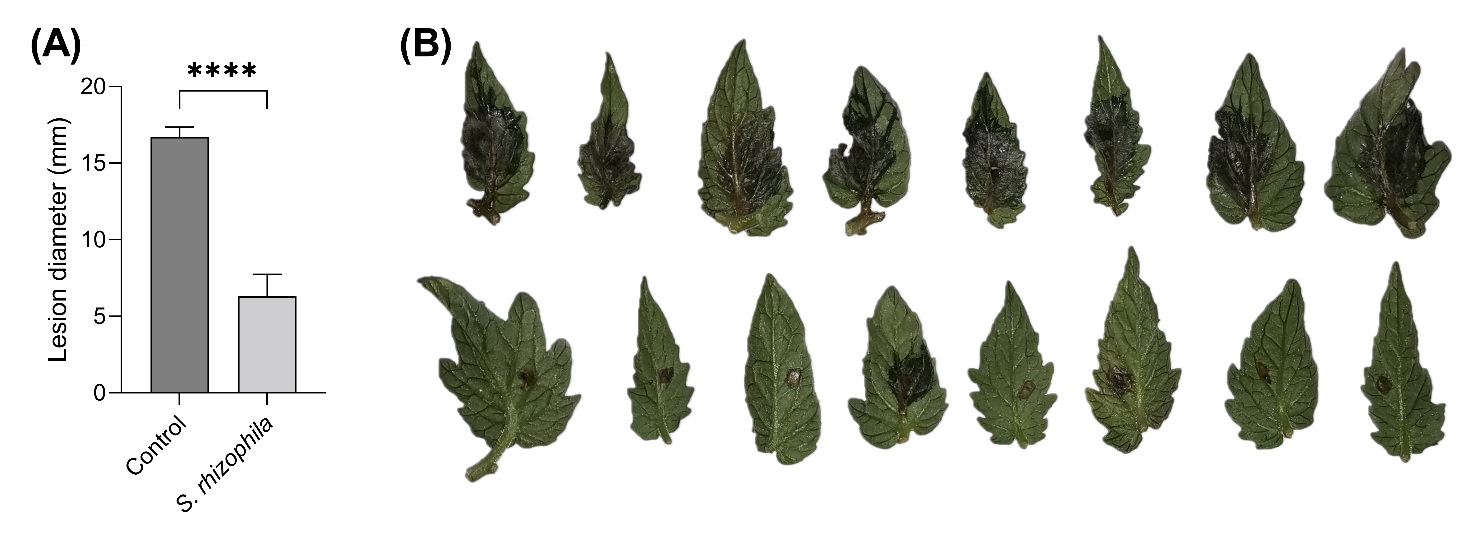
**

**Supplementary Figure S4**. *S. rhizophila* Ep2.2 protects whole tomato plants (cv. Marmande) from *B. cinerea* infection. Plants were treated with a bacterial suspension of *S. rhizophila* Ep2.2 and inoculated 48 hours later on two opposite leaves with a single 10-µL drop of 1×10^6^ *B. cinerea* conidia/mL. Lesions caused by *B. cinerea*  were measured after 3 days of incubation (mean ± SEM, *n* = 10-12) **(A)**. Asterisks indicate statistically significant differences at *p* < 0.001 (***). Pictures were taken on the same day. Control plants **(B)** and plants treated with *S. rhizophila* Ep2.2 **(C)**. Symptoms caused by *B. cinerea* in control and treated plants are clearly different (arrowheads).

**
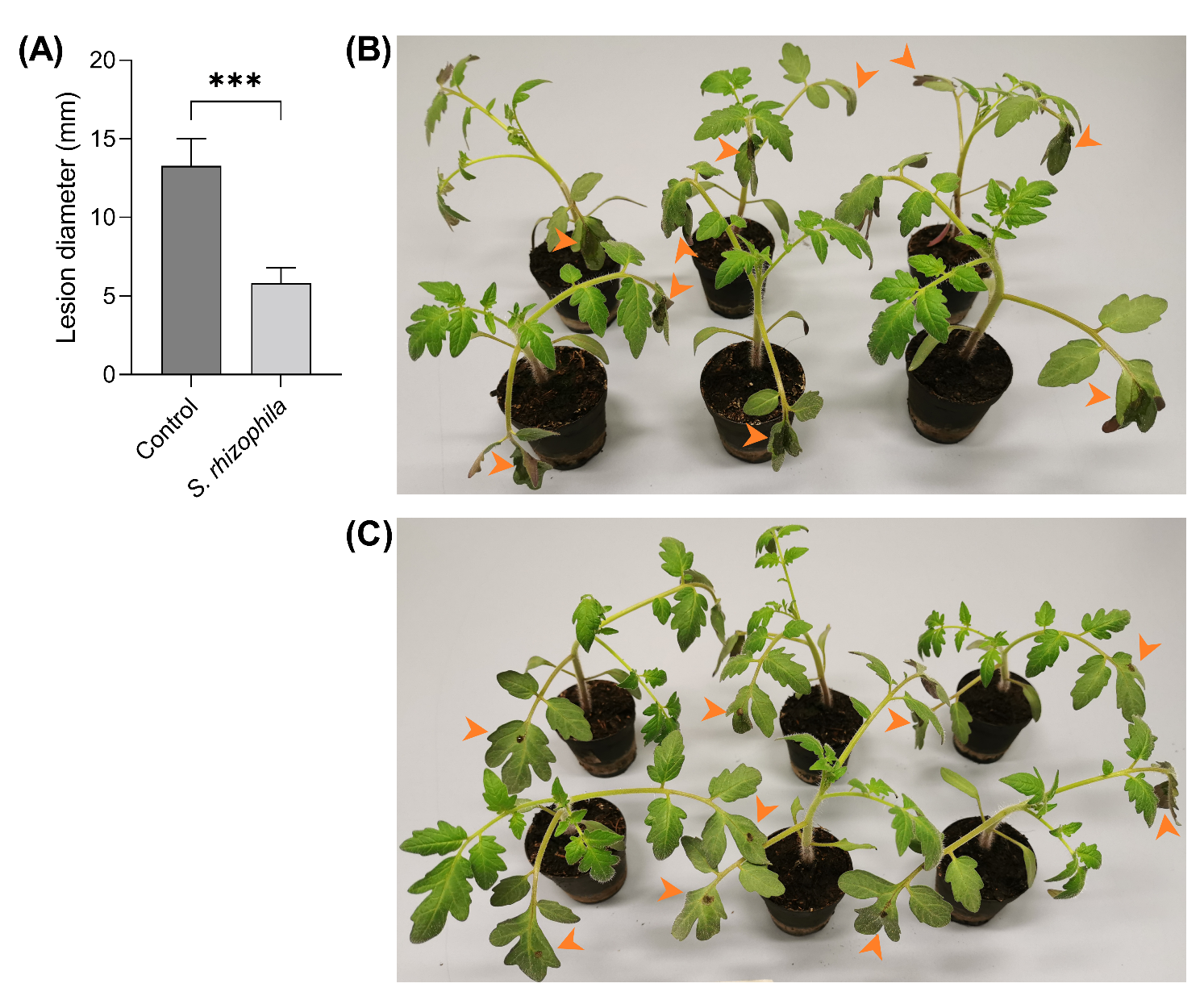
**

**Supplementary Figure S5**. Content of the Biolog GEN III MicroPlate used to compare the strain Ep2.2 of *S. rhizophila* with *S. malthophilia* strain LMG 958. The GEN III MicroPlate includes 94 phenotypic tests: 71 carbon source utilization assays (columns 1-9) and 23 chemical sensitivity assays (columns 10-12). The chemicals are prefilled and dried into the wells. Tetrazolium redox dyes are used to colorimetrically indicate utilization of the carbon sources or resistance to inhibitory chemicals.


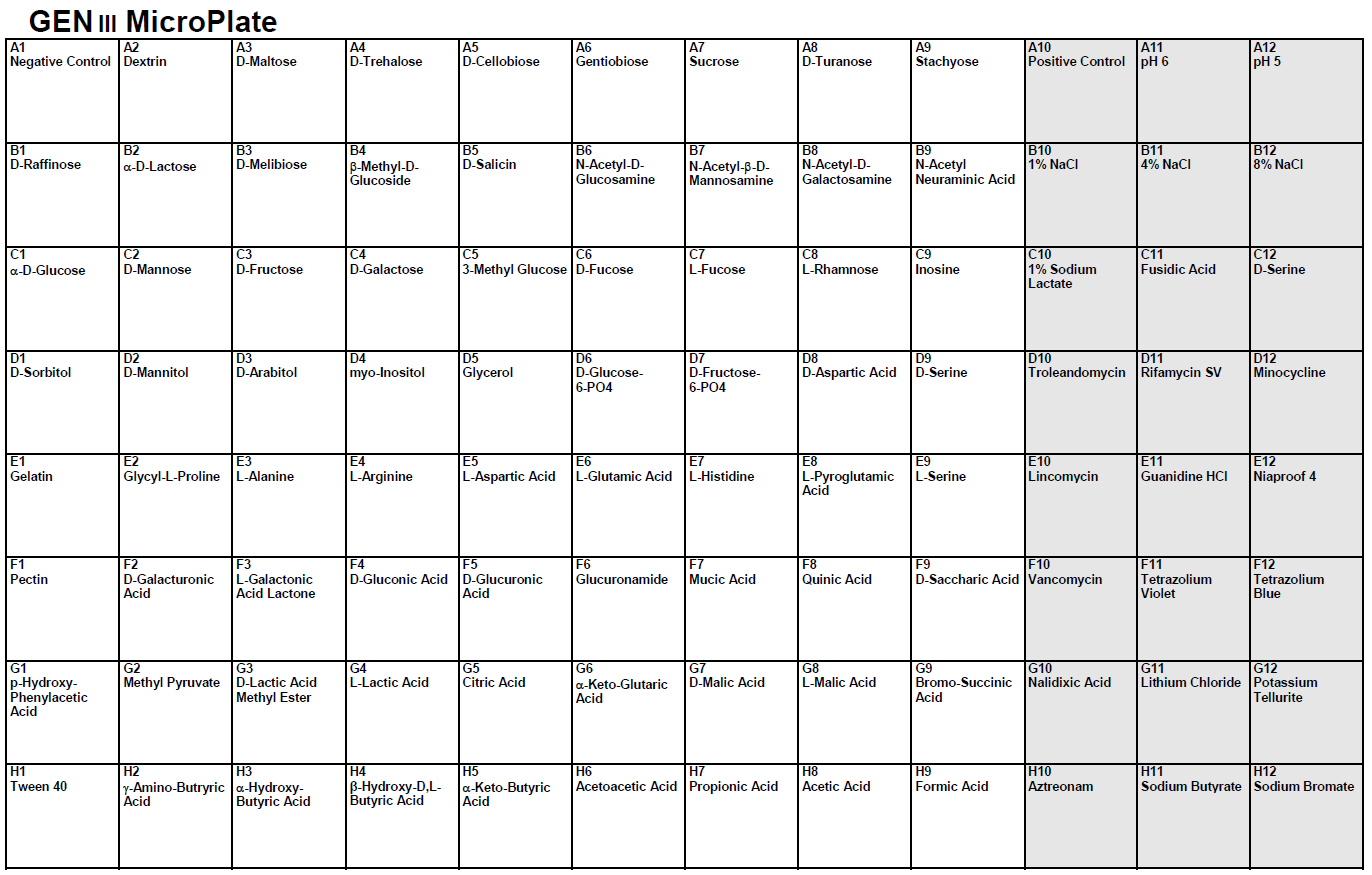


**Supplementary Table S1.** Bacterial strains used as reference for the *in vitro* tests. All the strains are included in the microbial collection of the Institute for Sustainable Plant Protection (IPSP-CNR, Florence, Italy).

| **Reference strain** | **Test** |
| --- | --- |
| *Agrobacterium tumefaciens* C58 | Catalase, oxidase, motility |
| *Bacillus* sp. B7 | KOH test |
| *Erwinia amylovora* E1 | Motility |
| *Pseudomonas chlororaphis* M71 | KOH test, production of lipases, proteases, IAA, siderophores |
| *Pseudomonas* sp. PK18 | Phosphate solubilization |
| *Serratia liquefaciens* CC7 | ACC deaminase, EPS production |
| *Stenotrophomonas malthophilia* LMG958 | Production of chitinase and β-glucanase, biofilm, motility |
| *Xanthomonas campestris* pv*. campestris* Xcc1 | Catalase, oxidase, motility |

**Supplementary Table S2 –** Protonated ions related to VOCs and/or fragments of VOCs emitted by *Stenotrophomonas rhizophila* Ep2.2 grown in axenic culture as detected by PTR-QMS analysis. The table is complementary to **Table 2** and includes the remaining ~ 2% of the blend detected by PTR-QMS. Mean values were calculated from 7-9 different bacterial cultures from two independent experiments and indicate the percentage of VOC on the total protonated ions detected, whereas ± errors express, in percentage, the standard error of the different replicates with respect to their raw mean values, before raking. n.a., not assigned.

| **Protonated ion (m/z)** | **Relative amount within the VOC blend (%) ± error (%)** | **Assignment to specific VOCs or fragments of VOCs** |
| --- | --- | --- |
| 50 | 0.39 ± 14.6 | n.a. |
| 113 | 0.28 ± 145.7 | e.g. heptenal, heptenone, octane, dimethyfuranone |
| 68 | 0.28 ± 34.7 | n.a. |
| 83 | 0.26 ± 744.1 | hexanals fragment |
| 109 | 0.18 ± 292.9 | e.g. pyrazine, 2,5-dimethyl- |
| 100 | 0.17 ± 86.3 | n.a. |
| 141 | 0.13 ± 77.1 | e.g. nonenal, nonenone |
| 64 | 0.08 ± 53.9 | n.a. |
| 137 | 0.07 ± 307.7 | monoterpenes |
| 115 | 0.06 ± 165.7 | e.g. heptan-2-one |
| 133 | 0.06 ± 36.2 | n.a. |
| 77 | 0.05 ± 242.1 | n.a. |
| 161 | 0.04 ± 47.2 | n.a. |
| 105 | 0.02 ± 330.4 | e.g. 1,2-pentanediol |
| 76 | 0.02 ± 155.7 | n.a. |
| 117 | 0.01 ± 275.8 | e.g. heptan-1-ol |
| 142 | 0.01 ± 183.8 | n.a. |
| 150 | 0.01 ± 100.7 | n.a. |

**Supplementary Table S3** – VOCs detected and identified in axenic cultures of *Stenotrophomonas rhizophila* Ep2.2 by GC-MS analysis. Mean values were calculated from 7-9 different bacterial cultures from two independent experiments and indicate the percentage of single VOCs on the whole blend of VOCs detected, whereas ± standard errors express the percentage of variation with respect to the mean values.

| **Chemical family** | **VOC name** | **Formula** | **Relative amount within the VOC blend (%)** |
| --- | --- | --- | --- |
| ALCOHOL | 2-METHYL PROPANOL | C4H10O | 0.5 ± 4.9 |
| ALDEHYDE | 2 METHYL PROPANAL | C4H8O | 1.2 ± 19.7 |
| ALDEHYDE | NONANAL | C9H18O | < 0.1 |
| ALDEHYDE | DECANAL | C10H20O | < 0.1 |
| ALKANE | 2 METHYL PENTANE | C6H14 | 4.2 ± 10.9 |
| ALKANE | N-HEXANE | C6H14 | 0.3 ± 11.4 |
| ALKANE | 2 METHYL HEPTANE | C8H18 | 0.1 ± 9.8 |
| ALKANE | 2,3 DIMEHTYL HEXANE | C8H18 | 0.5 ± 10.3 |
| ALKANE | 2,4 DIMEHTYL HEXANE | C8H18 | 0.2 ± 9.6 |
| ALKANE | 3 METHYL HEPTANE | C8H18 | < 0.1 |
| ALKANE | OCTANE | C8H18 | < 0.1 |
| ALKANE | 2,3,5 TRIMETHYL HEXANE | C9H20 | 1.4 ± 11.4 |
| ALKANE | 2,4 DIMETHYL HEPTANE | C9H20 | 9.5 ± 8.8 |
| ALKANE | 2,3 DIMETHYL HEPTANE | C9H20 | 1.7 ± 10.9 |
| ALKANE | 4 METHYL OCTANE | C9H20 | 8.0 ± 9.1 |
| ALKANE | 2,4,6 TRIMETHYL HEPTANE | C10H22 | < 0.1 |
| ALKANE | DECANE | C10H22 | 0.3 ± 11.9 |
| ALKANE | 3, 3 DIMETHYL OCTANE | C10H22 | 0.1 ± 13.9 |
| ALKANE | 2,4,6 TRIMETHYL OCTANE | C11H24 | 0.1 ± 12.4 |
| ALKANE | UNKNOWN ALKANE | C11H24 (tentative) | 0.2 ± 11.9 |
| ALKANE | UNDECANE | C11H24 | 4.2 ± 7.0 |
| ALKANE | 2 METHYL 6 ETHYL OCTANE | C11H24 | 1.0 ± 9.9 |
| ALKANE | UNKNOWN ALKANE | C11H24 (tentative) | 0.5 ± 9.3 |
| ALKANE | UNKNOWN ALKANE | C11H24 (tentative) | 0.1 ± 6.9 |
| ALKANE | 2,3,6,7 TETRAMETHYL OCTANE | C12H26 | 0.5 ± 9.3 |
| ALKANE | UNKNOWN ALKANE | C12H26 (tentative) | 0.4 ± 9.2 |
| ALKANE | 2,4 DIMETHYL DECANE | C12H26 | 5.2 ± 7.4 |
| ALKANE | 4,7 DIMETHYL UNDECANE | C12H28 | 2.0 ± 8.1 |
| ALKANE | 2, 9 DIMETHYL DECANE | C12H26 | 0.1 ± 34.4 |
| ALKANE | 5,7 DIMETHYL UNDECANE | C12H28 | 0.2 ± 10.7 |
| ALKANE | DODECANE | C12H26 | 1.5 ± 10.5 |
| ALKANE | UNKNOWN ALKANE | C12H26 (tentative) | 1.9 ± 7.8 |
| ALKANE | UNKNOWN ALKANE | C12H26 (tentative) | 3.5 ± 9.0 |
| ALKANE | UNKNOWN ALKANE | C12H26 (tentative) | 0.6 ± 6.4 |
| ALKANE | UNKNOWN ALKANE | C12H26 (tentative) | 2.3 ± 6.4 |
| ALKANE | 2,4, DIMETHYL UNDECANE | C13H28 | 0.1 ± 10.7 |
| ALKANE | 5 METHYL DODECANE | C13H28 | 0.7 ± 9.1 |
| ALKANE | 2,5,6 TRIMETHYL DECANE | C13H28 | 0.2 ± 10.1 |
| ALKANE | 3, 7 DIMETHYL UNDECANE | C13H28 | 0.2 ± 12.4 |
| ALKANE | UNKNOWN ALKANE | C13H28 (tentative) | 0.5 ± 5.8 |
| ALKANE | UNKNOWN ALKANE | C13H28 (tentative) | 0.5 ± 9.8 |
| ALKANE | UNKNOWN ALKANE | C13H28 (tentative) | 0.9 ± 5.2 |
| ALKANE | 4,8 DIMETHYL UNDECANE | C13H28 | 0.5 ± 7.1 |
| ALKANE | TRIDECANE | C13H28 | 1.0 ± 9.3 |
| ALKANE | 2,3,5,8 TETRAMETHYL DECANE | C14H30 | 0.2 ± 7.4 |
| ALKANE | TETRADECANE | C14H30 | < 0.1 |
| ALKANE | 4,6 DIMETHYL DODECANE | C14H30 | < 0.1 |
| ALKANE | 2, 6, 11 TRIMETHYL DODECANE | C15H32 | 0.7 ± 15.9 |
| ALKANE | HEXADECANE | C16H34 | 0.3 ± 8.0 |
| ALKANE | UNKNOWN ALKANE | C16H34 (tentative) | 0.3 ± 5.7 |
| ALKANE | UNKNOWN ALKANE | C16H34 (tentative) | 0.3 ± 18.3 |
| ALKENE | 2 BUTENE | C4H8 | 1.8 ± 18.7 |
| ALKENE | 4-METHYL-1-HEPTENE | C7H14 | 0.2 ± 11.6 |
| ALKENE | 2,4 DIMETHYL HEPTENE | C9H18 | 1.0 ± 10.3 |
| ALKENE | 2,4 DIMETHYL 1 DECENE | C12H24 | 0.2 ± 12.6 |
| ALKENE | 4, 6, 8 TRIMETHYL 1 NONENE | C12H24 | 0.2 ± 6.2 |
| ARENE | BENZENE | C6H6 | < 0.1 |
| ARENE | TOLUENE | C7H8 | < 0.1 |
| ARENE | MESITYLENE | C9H12 | 0.2 ± 25.7 |
| CARBOXYLIC ACID | BENZOIC ACID | C7H6O2 | < 0.1 |
| FURANE | FURAN | C4H4O | 2.4 ± 8.4 |
| FURANE | 2-4 DIMETHYL FURAN | C6H8O | 3.0 ± 31.2 |
| HALOALKANE | TRICHLORO METHANE | CHCl3 | 26.0 ± 15.7 |
| KETONE | ACETONE | C3H6O | 2.6 ± 18.3 |
| ORGANOSULFUR | DIMETHYL SULFIDE | C2H6S | 0.2 ± 30.8 |
| ORGANOSULFUR | DIMETHYL DISULFIDE | C2H6S2 | 3.2 ± 8.9 |
| TERPENE | α-PINENE | C10H16 | < 0.1 |
| TERPENE | CAMPHENE | C10H16 | < 0.1 |
| TERPENE | β-PINENE | C10H16 | < 0.1 |
| TERPENE | Δ-3-CARENE | C10H16 | < 0.1 |
